# MAPK20-mediated ATG6 phosphorylation is critical for pollen development in *Solanum lycopersicum* L.

**DOI:** 10.1093/hr/uhae069

**Published:** 2024-03-06

**Authors:** Yu Wang, Dongling Xie, Xuelian Zheng, Mingyue Guo, Zhenyu Qi, Ping Yang, Jingquan Yu, Jie Zhou

**Affiliations:** Department of Horticulture, Zhejiang University, Hangzhou 310058, China; Department of Horticulture, Zhejiang University, Hangzhou 310058, China; Department of Horticulture, Zhejiang University, Hangzhou 310058, China; Department of Horticulture, Zhejiang University, Hangzhou 310058, China; Hainan Institute, Zhejiang University, Sanya 572000, China; Agricultural Experiment Station, Zhejiang University, Hangzhou 310058, China; Agricultural Experiment Station, Zhejiang University, Hangzhou 310058, China; Department of Horticulture, Zhejiang University, Hangzhou 310058, China; Hainan Institute, Zhejiang University, Sanya 572000, China; Zhejiang Provincial Key Laboratory of Horticultural Plant Integrative Biology, Hangzhou 310058, China; Key Laboratory of Horticultural Plants Growth, Development and Quality Improvement, Ministry of Agriculture and Rural Affairs, Hangzhou 310058, China; Department of Horticulture, Zhejiang University, Hangzhou 310058, China; Hainan Institute, Zhejiang University, Sanya 572000, China; Shandong (Linyi) Institute of Modern Agriculture, Zhejiang University, Linyi 276000, China; Zhejiang Provincial Key Laboratory of Horticultural Plant Integrative Biology, Hangzhou 310058, China; Key Laboratory of Horticultural Plants Growth, Development and Quality Improvement, Ministry of Agriculture and Rural Affairs, Hangzhou 310058, China

## Abstract

In flowering plants, male gametogenesis is tightly regulated by numerous genes. Mitogen-activated protein kinase (MAPK) plays a critical role in plant development and stress response, while its role in plant reproductive development is largely unclear. The present study demonstrated MAPK20 phosphorylation of ATG6 to mediate pollen development and germination in tomato (*Solanum lycopersicum* L.). *MAPK20* was preferentially expressed in the stamen of tomato, and mutation of *MAPK20* resulted in abnormal pollen grains and inhibited pollen viability and germination. MAPK20 interaction with ATG6 mediated the formation of autophagosomes. Liquid chromatography–tandem mass spectrometry (LC–MS/MS) analysis showed that ATG6 was phosphorylated by MAPK20 at Ser-265. Mutation of *ATG6* in wild-type (WT) or in *MAPK20* overexpression plants resulted in malformed and inviable pollens. Meanwhile, the number of autophagosomes in *mapk20* and *atg6* mutants was significantly lower than that of WT plants. Our results suggest that MAPK20-mediated ATG6 phosphorylation and autophagosome formation are critical for pollen development and germination.

## Introduction

In flowering plants, male reproductive development is a transient but complex process, which is critical for normal pollination and fertilization [1]. Pollen development consists of three stages: microsporogenesis, microgametogenesis, and pollen maturation, encompassing intricate material and energy metabolism processes [2, 3]. The development of pollen involves complex and dynamic changes in the expression of many genes, which are tightly controlled by genetic networks and physiological metabolism.

*DYSFUNCTIONAL TAPETUM 1* (*DYT1*) and *DEFECTIVE IN TAPETAL DEVELOPMENT AND FUNCTION 1* (*TDF1*) have been shown to regulate the function of tapetum and the development of pollen in Arabidopsis [4, 5]. *ABORTED MICROSPORES* (*AMS*) regulates several genes related to tapetum function and biosynthesis, including pollen wall pattering and flavonoid synthesis in Arabidopsis [6]. The ortholog of AMS in rice, TAPETUM DEGENERATION RETARDATION, regulates tapetum degradation by interacting with ETERNAL TAPETUM 1 [7]. *MALE STERILITY 1* (*MS1*) exhibits transient expression during callose decomposition to free microspores [8] and is essential for the formation of pollen wall and tapetum programmed cell death (PCD) in Arabidopsis [9]. Furthermore, MYB80 serves as a pivotal transcription factor in the regulation of tapetal PCD by directly targeting *GLOX1* (a glyoxal oxidase), *VANGUARD1* (a pectin methylesterase), and *UNDEAD* (an A1 aspartic protease) [10]. Despite the extensive research on the key factors involved in anther and pollen development and tapetum PCD [11, 12], there is still limited understanding of the regulation of signaling processes and the activities of each component at different developmental stages. Many aspects of these processes remain poorly implicit, indicating the need for further investigation and clarification to unravel the complexities of these processes.

The mitogen-activated protein kinase (MAPK) consists of three sequential kinases: MAPK, MAPK kinase (MKK), and MAPKK kinase (MAPKKK), which are essential for intracellular and extracellular signaling in plants [13]. In plants, MAPK cascades participate widely in signaling transduction during abiotic stresses, pathogens, and phytohormones [14]. In addition, more and more studies have shown that the MAPK signaling pathway mediates almost every stage of plant reproduction, such as gametogenesis, anther development, pollen development, ovule development, and pollen tube guidance [15, 16]. In Arabidopsis *mapk4* mutant plants, microsporoblasts fail to undergo meiotic cytokinesis, resulting in abnormal pollen grains [17]. Furthermore, Arabidopsis *MAPK6* mediates inflorescence, anther, and embryo development [18]. The *mapk6* mutants not only exhibit suppress anther development and reduce male fertility, but also contain embryos that rupture from the seed coat [18]. Downregulation of *MAPK7* or *MAPK20* in tomato inhibits the transformation of mononuclear microspores to mature pollen grains, resulting in abnormal pollen [19, 20].

Protein kinase and phosphatase play vital roles in signaling transduction *via* phosphorylation and dephosphorylation of substrate proteins, resulting in activation of defense response and developmental processes [21, 22]. MAPK phosphorylates serine/threonine to further regulate the downstream signal pathway [20]. Many transcription factors are directly phosphorylated by MAPK to participate in regulation of growth and defense response [13, 23]. In the immune responses of *Nicotiana benthamiana*, phosphorylation of WRKYs by MAPK enhances WRKY-dependent *RBOHB* expression and cell death [24]. In tobacco cells, phosphorylation of NtMAP65–1 by MAPK inhibits the activity of microtubule-associated proteins and promotes the progression of cytokinesis [25]. Arabidopsis WRKY34 is transiently phosphorylated by MAPK3/MAPK6 in the early stage of pollen development, but decreases the level of phosphorylation immediately before pollen maturation; whereas, mutations in the sites of MAPK phosphorylation impair the role of WRKY34 [26].

The pollen development is supported by active cellular metabolism. Therefore, the synthesis and degradation of cellular materials are required to maintain the normal function of germ cells [27, 28]. The ubiquitin (Ub)-proteasome system (UPS) and autophagy pathways are mainly responsible for protein quality control [29]. Many single short-lived, damaged, and misfolded proteins are broken down by the UPS [30, 31]. Meanwhile, as the housekeeping mechanism of eukaryotes, autophagy can degrade macromolecular protein aggregates and organelles, playing a crucial role in nutrient cycling, cellular waste management, and organelle quality control [29, 32]. The double-membrane structure of autophagosomes is formed in the cytosol and can engulf intracellular components and transfer to vacuoles for further degradation and reuse [33–35]. Previous studies have shown that autophagy is involved in regulation of tapetal cells degradation and pollen development [36, 37]. During meiosis and subsequent mitosis, autophagy is involved in the clearance of cytoplasm and organelles, leading to the formation of haploid gametocytes [36, 37]. Defection of autophagy-related (*ATG*) gene results in delaying flower, decreased pollen quality and viability in Arabidopsis [32, 36]. *ATG6* is predominantly expressed in mature pollen; pollen development in *atg6* mutant plants is not affected, but it inhibits pollen germination, which leads to male sterility in Arabidopsis [38–40]. ATG6, a core component of the class III phosphatidylinositol-3 kinase (PI3K) complex, interacts with vacuolar protein sorting 34 (VPS34) and 15 (VPS15) to mediate the production of phosphatidylinositol-3-phosphate (PI3P) [41, 42]. PI3P is necessary for autophagy and endocytosis [42, 43]. The absence of homozygous null mutants of *ATG6*, *VPS34*, or *VPS15* is attributed to the unsuccessful transmission of male gametophytes [38–40, 44–46]. It has been reported that autophagosome-like structures are detected in tapetum cells at the stage of uninucleate during pollen development in wild-type (WT) rice plants [47]. Meanwhile, this process is completely compromised in *ATG7*-knockout mutants, resulting in inhibiting anther dehiscence and PCD-mediated tapetum cell degradation, decreasing the accumulation of lipid and starch in mature pollen grains, reducing pollen viability and germination rate [47], indicating that autophagy is necessary for postmeiotic anther and pollen development.

In Arabidopsis, MAPK has been found to phosphorylate a variety of substrates, including transcription factors under different biological processes [13, 16, 23]. C-Jun N-terminal kinase, the homologous of MAPK in plants, is found to mediate the activation of autophagy in animal cells [48], but whether MAPK regulates autophagy in plants remains elusive. Here, we found that *MAPK20* was predominantly expressed in stamen and mutation of *MAPK20* resulted in pollen defects and inhibited germination. Further analysis revealed that the interaction between MAPK20 and ATG6 mediated autophagosomes formation. Pollen grains of *atg6* mutants were malformed and decreased viability and germination. In addition, ATG6 was phosphorylated by MAPK20, and knockout of *ATG6* in *MAPK20* overexpressing plants also inhibited pollen development, germination and autophagosomes formation. Our results demonstrate that MAPK20-mediated ATG6 phosphorylation regulates the formation of autophagosomes to affect pollen development.

## Results

### Functional analysis of MAPK20 in pollen development and germination

**Figure 1:**
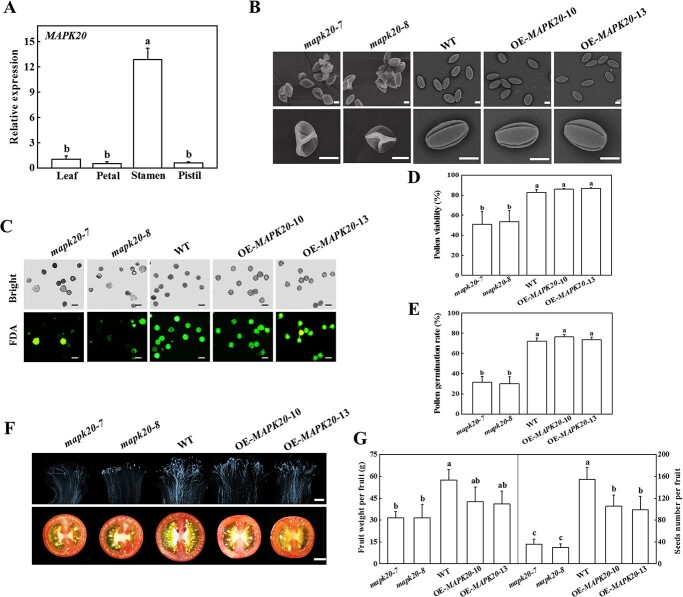
Functional analysis of *MAPK20* in pollen development and germination. **A** Relative expression levels of *MAPK20* in leaf and floral tissues (petal, stamen, and pistil) of tomato. **B** Scanning electron micrographs of mature pollen grains from WT, *MAPK20* gene knockout and overexpressing plants. Bars: 10 μm. **C** Fluorescein diacetate (FDA) staining detecting pollen viability of WT, *MAPK20* gene knockout and overexpressing plants. The viable pollen was stained green. Bars: 50 μm. **D** Pollen viability of WT, *MAPK20* gene knockout and overexpressing plants. **E** Pollen germination rate of WT, *MAPK20* gene knockout and overexpressing plants. **F** Aniline blue detecting pollen germination *in vivo* and the transverse sections of mature fruit of WT, *MAPK20* gene knockout and overexpressing plants. Bars: 50 μm (upper) and 1 cm (down). **G** Fruit weight and seed number per fruit from WT, *MAPK20* gene knockout and overexpressing plants. Results represent the means ± SD. Means with different letter showed significantly differ (*P* < 0.05). OE, overexpressing; WT, wild-type.

To analyse the function of *MAPK20* in tomato, we detected the expression levels of *MAPK20* in the leaf, petal, stamen, and pistil, and found that *MAPK20* was preferentially expressed in the stamen (Fig. 1A). The expression level of *MAPK20* in binucleate pollen (BP) and mature pollen (MP) stage was significantly higher than other stages (Fig. S1, see online supplementary material), suggesting that *MAPK20* might mediate pollen development. To analyse the role of *MAPK20* in pollen development, we generated two independent *mapk20* mutants using CRISPR/Cas9 technology. *mapk20–7* was 4-bp deletion in the second extron, and *mapk20–8* was a 1-bp insertion in the second extron (Fig. S2A–C, see online supplementary material). We also generated two independent *MAPK20* overexpressing (OE) lines (OE-*MAPK20*–10 and OE-*MAPK20*–13), which expressed a high level of MAPK20 protein (Fig. S2D, see online supplementary material). Pollen morphology of WT, *MAPK20* overexpressing, and knockout plants was observed using scanning electron microscopy (SEM). As shown in Fig. 1B, pollen grains of WT and *MAPK20* overexpressing plants were normal with oval shape and germination apertures distributed evenly. In contrast, a large number of adhesions of pollen grains in *mapk20* mutants appeared, which were malformed and collapsed (Fig. 1B). To accurately assess pollen development, we detected pollen viability of WT, *MAPK20* gene knockout, and overexpressing plants using fluorescein diacetate (FDA) staining. The results showed that the majority of pollen grains of WT and *MAPK20* overexpressing plants were viable, while, compared to WT plants, the pollen viability of *mapk20–7* and *mapk20–8* decreased by 38.3% and 35.1%, respectively (Fig. 1C and D). Only 31.4% and 29.9% pollens of *mapk20–7* and *mapk20–8* germinated, respectively, compared with 72.0% germinated pollens in WT plants (Fig. 1E; Fig. S3, see online supplementary material). However, the rate of pollen germination between WT and *MAPK20* overexpressing plants did not show any difference (Fig. 1E; Fig. S3, see online supplementary material). Consistent with the results in *vitro*, the germination rate of pollen grains of *mapk20* mutants *in vivo* decreased in comparison to WT plants (Fig. 1F). To test the occurrence of pollen abortion, we observed the microsporogenesis in tomato anther by semi-thin section in WT and *mapk20* mutant plants. Semi-thin section analysis revealed that the aborted pollen grains were observed in the BP and MP stage of *mapk20* mutants (Fig. S4, see online supplementary material).

Pollen grains deliver sperms to ovary to complete double fertilization, which is essential for fruit and seeds development [49]. To determine whether *MAPK20* was involved in fruit development, we compared the fruit weight and seed number between WT and *mapk20* mutant plants. The fruit weight of *mapk20* mutant plants was significantly reduced in comparison to WT plants (Fig. 1F and G). In addition, the seed number per fruit of *mapk20–7* and *mapk20–8* decreased by 77.0% and 80.8%, respectively, in comparison to WT plants (Fig. 1G). Thus, these results revealed that mutation of *MAPK20* affected pollen morphology, viability, germination, and fruit development.

### MAPK20 mediates the formation of autophagosomes

**Figure 2:**
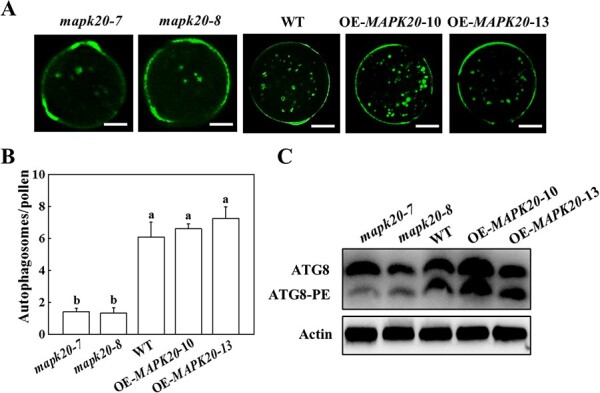
Analysis of autophagosomes in WT, *MAPK20* gene knockout and overexpressing plants. **A** MDC-stained autophagosomes in pollen grains of WT, *MAPK20* gene knockout and overexpressing plants. MDC-stained autophagosome is shown in green. Bars: 10 μm. **B** The number of MDC-stained autophagosomes in pollen grains. Results represent the means ± SD. Means with different letters showed significant difference (*P* < 0.05). **C** ATG8 protein levels in the anthers of WT, *MAPK20* gene knockout and overexpressing plants. Actin was used as a loading control. OE, overexpressing; WT, wild-type.

Autophagy plays vital roles in pollen germination in plants [50]. To test whether autophagy is involved in *MAPK20*-mediated pollen germination, we used monodansylcadaverine (MDC) staining to detect the formation of autophagosomes in tomato pollen grains. Fewer MDC-labeled fluorescence puncta were observed in the pollen grains of *mapk20* mutants, but their numbers in WT and *MAPK20* overexpressing pollen grains were significantly higher than of those observed in *mapk20* mutants (Fig. 2A and B). Furthermore, immunoblotting was used to detect the formation of ATG8-phosphatidylethanolamine (PE) with an anti-ATG8 antibody, which is widely used to monitor autophagosomes in plants [51]. As expected, the level of ATG8-PE in the anther of *mapk20* mutants was weak, but their levels in WT and *MAPK20* overexpressing anthers were higher than that in *mapk20* mutants (Fig. 2C), indicating that deficiency of *MAPK20* compromised autophagosmes formation.

### MAPK20 interaction with ATG6 mediates autophagosomes formation

**Figure 3:**
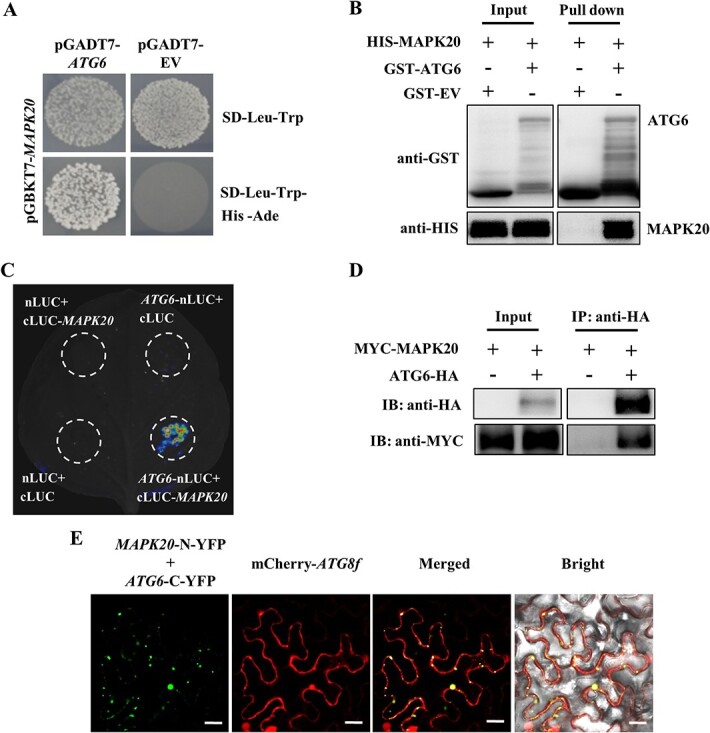
MAPK20 interaction with ATG6 regulating the formation of autophagosomes. **A** Yeast two-hybrid assays analysis MAPK20 interaction with ATG6 proteins. **B** Pull-down assay analysis the interaction of MAPK20 and ATG6. **C** Split-luciferase assay showing MAPK20 interaction with ATG6. *Agrobacterium tumefaciens* carrying the indicated constructs were co-infiltrated into the leaves of *Nicotiana benthamiana* and the chemiluminescence was detected after 2 d of inoculation. Empty vectors were used as the control. **D** Co-immunoprecipitation of MYC-MAPK20 and ATG6-HA proteins transiently co-expressed in *N. benthamiana* leaves. Total proteins were isolated and used for immunoprecipitation with anti-HA magnetic beads and immunoblotted with an anti-MYC or anti-HA antibody. **E** Bimolecular fluorescence complementation analysis of MAPK20 interaction with ATG6 and co-localization with ATG8f. Bars: 25 μm.

To investigate the potential mechanism of MAPK20 regulating autophagy, yeast two-hybrid assay was used to test whether MAPK20 interacted with ATG proteins. The CDS of *MAPK20* was introduced into the pGBKT7 vector. Besides, the CDS of another nine *ATG* genes were fused to the pGADT7 vector and transformed into yeast cells with pGBKT7-*MAPK20*. Only ATG6 was found to interact with MAPK20 (Fig. 3A; Fig. S5A). Furthermore, the interaction of MAPK20 and ATG6 was also observed in yeast when pGBKT7-*ATG6* and pGADT7-*MAPK20* were co-transformed into yeast cells (Fig. S5B, see online supplementary material). Subsequently, we made four truncations (ATG6–1 to ATG6–4) for ATG6 to further narrow the interaction sites. The results showed that ATG6–1 (1–83 aa of ATG6) and ATG6–4 (485–523 aa of ATG6) interacted with MAPK20 (Fig. S5C and D; see online supplementary material). Furthermore, GST pull-down assays indicated that only GST-tagged ATG6 was precipitated with the HIS-tagged MAPK20 (Fig. 3B), suggesting that GST-ATG6 directly interacted with HIS-MAPK20 protein *in vitro*.

To further test the interaction of MAPK20 with ATG6 *in vivo*, split-luciferase complementation assays were performed. As shown in Fig. 3C, a strong signal was observed in the leaves co-expressing of *ATG6*-nLUC and cLUC-*MAPK20*. Subsequently, the interaction between MAPK20 and ATG6 was confirmed with Co-immunoprecipitation (Co-IP) assay. The results showed that MYC-tagged MAPK20 was precipitated with HA-tagged ATG6, while the control experiment failed to precipitate the MYC-tagged MAPK20 protein (Fig. 3D). Furthermore, we detected their interaction in *N. benthamiana* leaves *via* bimolecular fluorescence complementation (BiFC) assay. BiFC signals were monitored with punctate fluorescence structurer in the leaves co-transformed with *MAPK20*-N-YFP and *ATG6*-C-YFP (Fig. 3E; Fig. S6, see online supplementary material), which might be the pre-autophagosome or autophagosomes structures. To verify MAPK20 interaction with ATG6 mediated the formation of autophagosomes, we used mCherry-tagged ATG8f as a marker of autophagosomes to test whether the BiFC signal could co-localize with mCherry-ATG8f signal. Interestingly, the fluorescence signal of MAPK20 interaction with ATG6 co-localized with mCherry-ATG8f signal (Fig. 3E). These results revealed that MAPK20 interacted with ATG6 to regulate the formation of autophagosomes.

**Figure 4:**
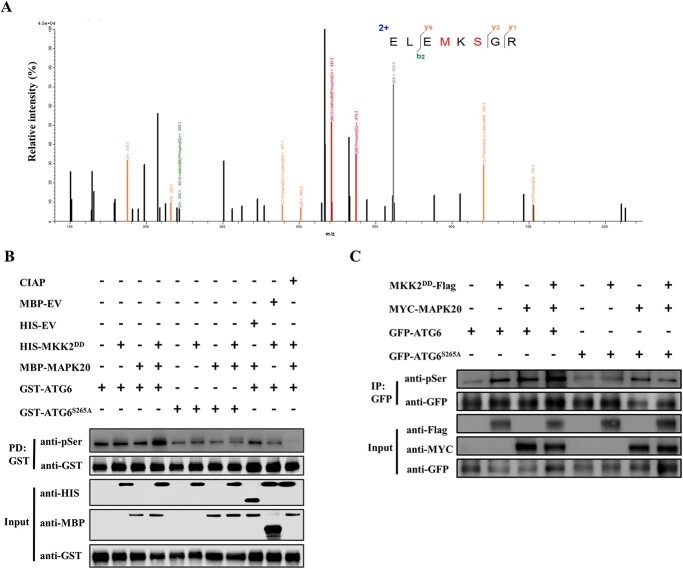
MAPK20 induces phosphorylation of ATG6 protein. **A** LC–MS/MS assay analysis MAPK20 phosphorylation of ATG6. The leaf samples of *Nicotiana benthamiana* co-expressing of MKK2^DD^-Flag, MYC-MAPK20, and ATG6-HA, or MYC-MAPK20 and ATG6-HA, or ATG6-HA proteins were collected after 2 d of infiltration, and the ATG6-HA protein-immunoprecipitated by anti-HA magnetic beads was sent for LC–MS/MS. **B** MAPK20 phosphorylates ATG6 in *vitro*. MBP-MAPK20 was activated using the upstream kinase HIS-MKK2^DD^ and phosphorylated using GST-ATG6 as a substrate, separated by SDS-PAGE and detected with an anti-pSer antibody. Calf intestinal alkaline phosphatase (CIAP) was used to confirm the phosphorylation of ATG6 protein. **C** MAPK20 induces the phosphorylation of ATG6 in *vivo*. MKK2^DD^-Flag, MYC-MAPK20, and GFP-ATG6 were co-expressed in *N*. *benthamiana* leaves for 2 d, then the protein was extracted and immunoprecipitated with GFP agarose beads. After separation by SDS-PAGE, phosphorylated ATG6 protein was detected by anti-pSer antibody. EV, empty vector.

To identify MAPK20-mediated ATG6 phosphorylation sites, we constructed constitutively activated MKK2 *via* mutation T215D and S221D according to the previously described method [52], to activate MAPK20. We collected leaf samples from co-expressing of ATG6-HA, MYC-MAPK20 and MKK2^DD^-Flag, or ATG6-HA and MYC-MAPK20, or ATG6-HA. Then, ATG6-HA proteins were immunoprecipitated and used for liquid chromatography–tandem mass spectrometry (LC–MS/MS) analysis. Only one serine residue (S265) on ATG6 protein in the samples of co-expressing of ATG6-HA, MYC-MAPK20, and MKK2^DD^-Flag was phosphorylated (Fig. 4A; Table S1, see online supplementary material). However, no phosphorylation residue on ATG6 protein was detected in the samples either co-expressing ATG6-HA and MYC-MAPK20 or only expressing of ATG6-HA (Table S1, see online supplementary material). These results indicated that MAPK20 phosphorylated ATG6 on S265. To assess whether MAPK20 induced phosphorylation of ATG6 protein, we performed phosphorylation reactions *in vitro*. In the presence of the upstream kinase HIS-MKK2^DD^, the MBP-MAPK20 fusion recombinant protein was shown to phosphorylate the GST-ATG6 substrate, based on immunoblotting with anti-pSer antibody, while the phosphorylated level of GST-ATG6^S265A^ was greatly decreased (Fig. 4B). Interestingly, the phosphorylation signal disappeared in the presence of calf intestinal alkaline phosphatase (CIAP) (Fig. 4B). Furthermore, when MKK2^DD^-Flag, MYC-MAPK20, and GFP-ATG6 were co-expressed in the leaves of *N*. *benthamiana*, the phosphorylation level of ATG6 was obviously increased (Fig. 4C). However, the phosphorylation level of ATG6^S265A^ was profoundly decreased (Fig. 4C).

### ATG6 is essential for pollen development

**Figure 5:**
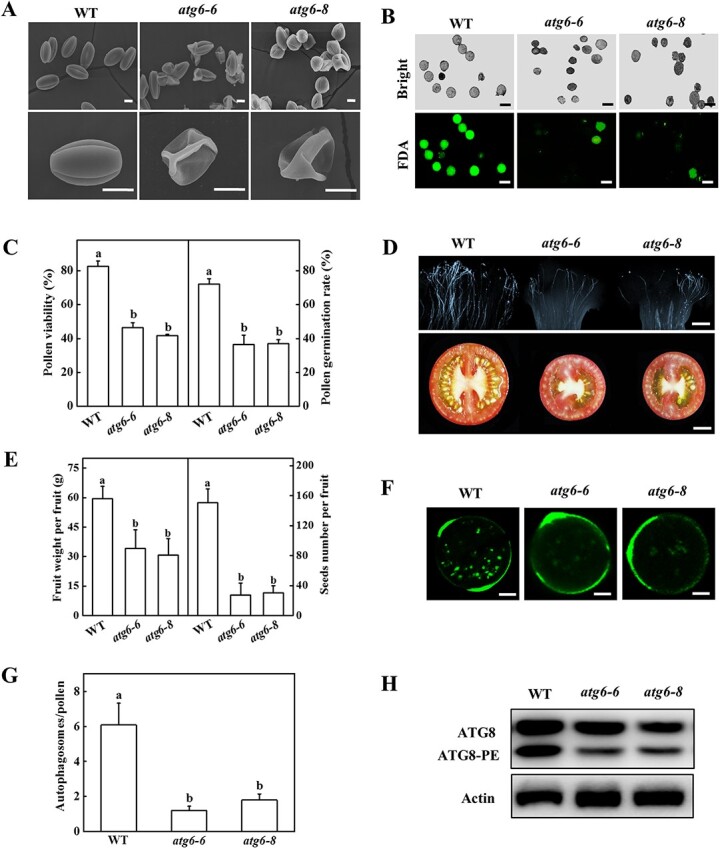
Functional analysis of *ATG6* in pollen germination and autophagosomes formation. **A** Scanning electron micrographs of mature pollen grains from the WT and *atg6* mutants. Bars: 10 μm. **B** Fluorescein diacetate (FDA) staining detecting pollen viability of WT and *atg6* mutant plants. The viable pollen was stained green. Bars: 50 μm. **C** Pollen viability and germination rate of WT and *atg6* mutant plants. **D** Aniline blue detecting pollen germination *in vivo* and the transverse sections of mature fruit of WT and *atg6* mutant plants. Bars: 50 μm (upper), 1 cm (lower). **E** Fruit weight and seed number per fruit of WT and *atg6* plants. **F** MDC-stained autophagosomes in the pollen grains of WT and *atg6* mutant plants. The MDC-stained autophagosome is shown in green. Bars: 10 μm. **G** The number of MDC-stained autophagosomes in **F**. **H** ATG8 protein levels in the anthers of WT and *atg6* mutant plants. Actin was used as a loading control. Results represent the means ± SD. Means with different letters showed significantly differ (*P* < 0.05). WT, wild-type.

To analyse the function of autophagy in pollen development, the expression of 26 *ATG* genes in leaf, petal, stamen, and pistil of tomato was examined. The expression levels of several *ATG* genes were upregulated in the petal and the majority of *ATG* gene expression was highly enhanced in the stamen in comparison to other tissues (Fig. S7A, see online supplementary material). Furthermore, the expression level of most of the *ATG* genes gradually enhanced with the development of pollen, especially in the BP and MP stage (Fig. S7B, see online supplementary material), suggesting that autophagy might play an essential role in pollen development. Thus, we constructed *atg6* mutant plants using CRISPR/Cas9 technology to detect its function in pollen development. Two independent *atg6* lines, *atg6–6* and *atg6–8* were mutant at the 3rd base of the protospacer adjacent motif (PAM) (Fig. S8, see online supplementary material). SEM observation showed that pollen grains of *atg6* mutant plants were partially defective (Fig. 5A). Semi-thin section observation found that partial pollen grains in the BP and MP stage of *atg6* mutants were abortion (Fig. S9, see online supplementary material). Furthermore, the expression of *TDF1*, *MS1-like*, and *MYB80* in *atg6* mutants was significantly decreased compared with WT plants (Fig. S10, see online supplementary material). To further verify the function of *ATG6* in pollen, we measured the pollen vitality by FDA staining. FDA staining showed that the pollen viability of *atg6* mutants was significantly reduced in comparison to WT plants (Fig. 5B and C). The results of pollen germination *in vitro* were in accord with pollen viability. The germination rate of *atg6–6* and *atg6–8* mutants decreased by 49.2% and 48.6%, respectively, compared with that observed in WT (Fig. 5C; Fig. S11, see online supplementary material). Subsequently, we evaluated the pollen germination on the stigma by hand pollination. Numerous germinated pollen tubes were observed in WT plants, whereas the germination of the pollens in *atg6* mutants was scarce (Fig. 5D). Furthermore, the fruit weight of *atg6–6* and *atg6–8* decreased by 42.5% and 48.4%, respectively, compared with WT (Fig. 5D and E). The seed numbers per fruit of *atg6–6* and *atg6–8* were only 17.7% and 19.7% of WT plants, respectively (Fig. 5D and E). These results indicated that *ATG6* was essential for pollen viability and germination.

To test the role of *ATG6* in pollen autophagy, we used MDC staining and immunoblotting to monitor the autophagosomes in the pollen grains of WT and *ATG6* gene knockout plants. The number of MDC-stained fluorescence puncta in WT pollen grains was significantly higher than that of observed in *atg6* mutant pollen grains (Fig. 5F and G). Similarly, the abundance of ATG8-PE of WT plants was more evident than that in *atg6* mutants (Fig. 5H).

To investigate whether PI3P mediated the pollen development and germination of *MAPK20* and *ATG6*, we analysed PI3P content in the leaves of WT, *mapk20*, and *atg6* mutants using a PI(3)P Mass ELISA Kit that is widely used to measure PI3P content [53, 54]. The content of PI3P in WT leaves did not display a significant difference with *mapk20* and *atg6* mutants (Fig. S12A, see online supplementary material). Pollen germination rate was detected on the medium in the presence of PI3P and its carrier, which forms a complex with PI3P to facilitate its transportation into cells [45]. The pollen germination rate of WT decreased by 11.6% on the medium supplied with PI3P and carrier in comparison to the medium containing only carrier (Fig. S12B and C, see online supplementary material). However, exogenous treatment with PI3P and carrier promoted pollen germination of *mapk20–7*, *mapk20–8*, *atg6–6*, and *atg6–8*, as indicated a notable increase of 62.9, 76.9, 52.1, and 59.1%, respectively, compared with their pollens on medium containing only the carrier (Fig. S12B and C, see online supplementary material).

### ATG6 is involved in MAPK20-mediated pollen germination

**Figure 6:**
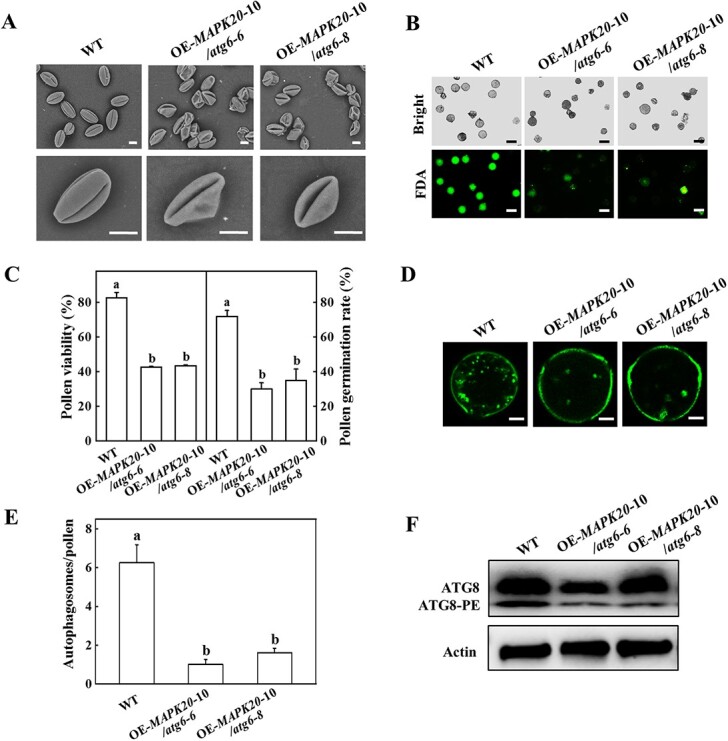
Functional analysis of *ATG6* in *MAPK20*-mediated pollen germination and autophagosomes. **A** Scanning electron micrographs of mature pollen grains from the WT and OE-*MAPK20/atg6* plants. Bars: 10 μm. **B** Fluorescein diacetate (FDA) staining detecting pollen viability of WT and OE-*MAPK20/atg6* plants. The viable pollen was stained green. Bars: 50 μm. **C** Pollen viability and germination rate of WT and OE-*MAPK20/atg6* plants. **D** MDC-stained autophagosomes in the pollen grains of WT and OE-*MAPK20/atg6* plants. MDC-stained autophagosome is shown in green. Bars: 10 μm. **E** The number of MDC-stained autophagosomes in **D**. **F** ATG8 protein levels in the anthers of WT and OE-*MAPK20/atg6* plants. Actin was used as a loading control. Results represent the means ± SD. Means with a different letter showed significant difference (*P* < 0.05). OE, overexpressing; WT, wild-type.

To test the function of *ATG6* in MAPK20-mediated pollen germination, we analysed pollen morphology, viability, germination, and autophagosomes in OE-*MAPK20*–10/*atg6* plants. The majority of pollen grains in OE-*MAPK20*–10/*atg6–6* and OE-*MAPK20*–10/*atg6–8* plants were aborted or defective (Fig. 6A). The pollen viability and germination rate of OE-*MAPK20*–10/*atg6–6* and OE-*MAPK20*–10/*atg6–8* plants were significantly lower than that in WT plants (Fig. 6B and C; Fig. S13, see online supplementary material). Furthermore, MDC-stained fluorescence puncta were obviously observed in WT pollen grains, but they were barely detected in OE-*MAPK20*–10/*atg6–6* and OE-*MAPK20*–10/*atg6–8* pollen grains (Fig. 6D and E). The band of ATG8-PE in WT plants was more evident than that in OE-*MAPK20*–10/*atg6–6* and OE-*MAPK20*–10/*atg6–8* plants (Fig. 6F). These results suggested that *ATG6* was involved in MAPK20-mediated pollen germination.

## Discussion

In flowering plants, the development of pollen is regulated by numerous genes, and is a complex process. MPAK signaling pathway is necessary in plant reproductive development [17, 26]. Meanwhile, the role of autophagy in plant growth and development has received rigorous attention recently. Studies have shown that loss of function of either *MAPKs* or *ATGs* results in impaired pollen fertility [15, 29, 37]. However, whether there is any relationship between MAPK and autophagy in plants and how they interact is still unclear. In this study, we demonstrated that MAPK20 phosphorylates ATG6 to mediate autophagosomes formation to regulate tomato pollen development and germination. These results provide new evidence for the important roles of MAPK20 and ATG6 in the reproductive development of tomato.

MAPKs remain active at different stages of plant reproductive development, including pollen development and germination, pollen tube guidance, and ovule development [13, 15]. *mapk3^+/−^mapk6^−/−^* mutant plants have abnormal integuments due to inhibition of late cell division, which inhibits embryo sac development and leads to female sterility [55]. *mapk3mapk6* double mutants are embryonic lethal and impair pollen tube guidance [13]. In *Papaver rhoeas*, *PrMAPK9–1* is mainly expressed in pollen and is involved in regulating self-incompatibility [56]. The expression of Ntf4, a MAPK protein in tobacco, is gradually enhanced during pollen development, especially in pollen maturation and germination [57]. Moreover, Ntf4 protein transfers from cytoplasm to the nucleus at late pollen developmental stages [57]. Furthermore, silencing or knockout of tomato *MAPK20* largely arrests pollen development at the BP stage with the appearance of subcellular abnormality, and significantly decreases pollen viability [20]. Ultrastructural observation found that the nuclei and cytoplasmic content breaks down at the BP stage of *mapk20* mutants, and the mature pollens are collapsed [20]. Pollination of *mapk20* mutant flowers with WT pollen grains yields normal seed and fruit development, indicating that knockout of *MAPK20* resulting in sterility is entirely due to male rather than female deficiency [20]. A similar phenotype is observed in *MAPK4* or *MAPK7* silenced tomato plants [19, 58]. A total of 16 *MAPK* genes are identified in tomato, and they can be divided into four distinct groups [59]. Tomato MAPK4 and MAPK7 belong to the MAPK group B, while MAPK20 is a member of group D, which is distinctly separated from the other three groups [59]. Kong *et al.* [59] analysed the expression profiles of the *MAPKs* in stamen of tomato, and found that except for *MAPK9*, other *MAPKs* had higher expression levels in stamens than in other organs, suggesting that MAPKs might have conserved functions during reproduction. Consistent with previous studies, we found that *MAPK20* was preferentially expressed in stamens of tomato (Fig. 1A). Further genetic analysis showed that deficiency of *MAPK20* resulted in abnormal morphology of mature pollen, decreased pollen vigor and germination rate, and correspondingly significantly reduced fruit weight and seed number (Fig. 1). Although overexpressing *MAPK20* did not show significant difference in the pollen morphology and germination, the fruit weight and seeds number of *MAPK20* overexpressing plants were lower than those in WT plants (Fig. 1). Similarly, *MAPK4* overexpressing plants do not affect pollen morphology and germination, but the fruit set frequency is significantly lower than that in WT plants [58]. Furthermore, overexpressing of *MAPK7* also slightly reduces the number of seeds [19]. *MAPK4* RNAi plants display over 10% parthenocarpic fruits, but WT plants cannot obtain fruit without pollination [58]. These results suggest that *MAPKs* might affect other pathways of seed and fruit development. Therefore, it is imperative to conduct additional research to elucidate the specific mechanisms involved in this process.

Autophagy, an evolutionary conserved mechanism for self-degradation in eukaryotic cells, is required for anther metabolic regulation and nutrient supply [32, 36]. Autophagy-deficient plants inhibit the formation of autophagosomes, delay flowering, and decrease pollen germination [37, 60]. Rice *atg7* knockout mutants exhibit male sterility, restricted anther dehiscence, and difficulty in lipid and starch accumulation in pollen grains [47]. Furthermore, Arabidopsis *Beclin1*, the tomato *ATG6* orthologue, has been shown to be preferentially expressed in mature pollen grains, and pollen germination is severely inhibited in *Beclin1*-deficient plants [38, 39]. In this study, we found that *ATG6* was predominately expressed in stamen of tomato (Fig. S7A, see online supplementary material). The pollen grains of *atg6* mutant plants were malformed and their viability and germination were significantly lower than that of WT (Fig. 5). Additionally, the *ATG6* mutant allele cannot be transmitted by male gametophytes and homozygous *ATG6* mutants are embryo lethal in Arabidopsis, indicating that ATG6 is essential for development. Indeed, the *atg6–2* heterozygotes showed a difference in phenotypes when compared to WT plants, such as retarded growth and decreased the number of silique [40]. Similarly, Arabidopsis *ATG6* antisense plants displayed a severely stunted phenotype, including smaller leaves length and area, thinner inflorescence stems, and fewer flowers, siliques, and seeds [61]. Furthermore, *ATG6* antisense plants increased sensitivity to nutrient starvation due to reducing the formation of autophagosomes [61]. These obvious stunted phenotypes were not observed in tomato *atg6* mutant plants under normal growth conditions, but inhibited autophagic activity and increased sensitivity to low-nitrogen stress [62]. These results suggested that tomato *ATG6* may have different functional mechanisms in development with that in Arabidopsis. ATG6, as a core member of PI3K, mediates the production of PI3P, and PI3P plays an important role in Arabidopsis pollen development [42]. The homozygous mutants of *ATG6*, or *VPS34*, or *VPS15* are lethal in Arabidopsis, and their heterozygotes also significantly inhibit pollen germination due to lack of PI3P, suggesting that the PI3K complex-mediated PI3P is essential for Arabidopsis [39, 44, 45]. VPS38 or ATG14 acts as the fourth subunit of PI3K complex, but the triple mutants of *atg14aatg14bvps38* are viable with approximately 50% accumulated PI3P content compared with WT plants [43], indicating that the PI3K complex plays a decisive role in the synthesis of PI3P in Arabidopsis. However, knockout *ATG6* displayed no significant effect on PI3P content in tomato leaves (Fig. S12A, see online supplementary material), suggesting that ATG6 might not be essential or act as auxiliary roles for PI3P synthesis, or there is a novel PI3K type which is independent in class III PI3K in tomato.

MAPKs play vital roles in the process of pollen development and germination by phosphorylation of downstream substrates [63, 64]. MAPK3 and MAPK6 phosphorylate downstream WRKY2 and WRKY34 in pollen development and are involved in starch and/or fatty acid biosynthesis and liposome accumulation during pollen maturation [26, 65]. Exo70A1, a stigma receptivity factor, is phosphorylated by MAPK3 and MAPK4 to induce its localization to the plasma membrane, which is essential for regulating exocytosis for pollination [63]. In addition, study of Arabidopsis reveals a critical role for TYPE ONE PROTEIN PHOSPHATASE in regulating autophagy by dephosphorylating ATG13a to stimulate the formation of the ATG1a-ATG13a complex [66]. Here, we demonstrated that MAPK20 interacted with ATG6 to mediate autophagosomes formation (Fig. 3), while *mapk20* and *atg6* mutants blocked autophagosomes formation, resulting in malformed mature pollen and impaired fertility (Figs 1, 2, and 5). In addition, ATG6 was phosphorylated by MAPK20 (Fig. 4). Knockout of *ATG6* gene in *MAPK20* overexpressing plants not only compromised the formation of autophagosomes but also significantly inhibited pollen germination (Fig. 6), suggesting that ATG6-mediated autophagosome formation acts as a downstream of MAPK20 and affects pollen maturation and germination.

Transcriptomic and proteomic analyses revealed a dramatic decrease of protein synthesis at late stages of pollen development, with gene expression biased towards cell wall metabolism, cytoskeleton and signaling, in preparation for processes including identification of target tissues and rapid directional growth of pollen tubes [67, 68]. The expression patterns of various organelle- and carbohydrate/lipid metabolism-related genes are also affected during pollen maturation in rice *atg7–1* mutants [32]. In addition, silencing of the *ATG* gene reduces autophagic activity and fails to degrade the convex cytoplasmic layer at the germination hole, thereby inhibiting pollen germination [50], indicating that autophagy is also actively involved in pollen germination. In this study, we found that the knockout *ATG6* gene resulted in partial defective pollen, inhibiting the formation of autophagosomes, and decreasing germination rate (Fig. 5C and G). Furthermore, *mapk20* mutants and knockout *ATG6* in *MAPK20* overexpressing plants resulted in impaired autophagosome formation and germination (Figs. 1 and 6). Thus, MAPK20 modulated pollen development and germination through autophagy. In addition, *MAPK20* modulates pollen development through regulating sugar and auxin metabolism and signaling [20]. Therefore, as a kinase, MAPK20 may regulate pollen development through various pathways.

To sum up, we provided novel insights into the critical role of MAPK20 and ATG6 in pollen development. *MAPK20* was predominantly expressed in stamen and mutation of *MAPK20* resulted in a pollen defect and inhibited germination. MAPK20 phosphorylated ATG6 to mediate the formation of autophagosomes, which affected pollen maturation and germination.

## Materials and methods

### Plant materials and growth conditions

Tomato (Ailsa Craig) was used in all of the experiments. Tomato plants were irrigated with 1/2 Hoagland nutrition solution every 3 d. The growth conditions were controlled at 25/20°C (day/night) with a photoperiod of 14 h, 600 μmol m^−2^ s^−1^ photosynthetic photon flux density (PPFD). The five-leaf expanded tomato seedlings were transferred into a 4 L plastic pot and cultivated in the greenhouse. The air temperature was controlled at 28 ± 1°C /19 ± 1°C (day/night), with a maximum PPFD of 1000 μmol m^−2^ s^−1^ and relative humidity of 50–65%.

### Total RNA isolation and gene expression analysis

Total RNAs were isolated from different tissues of tomato, reverse transcribed to cDNA, and the qPCR assays were conducted and relative gene expression was calculated as previously described [69]. Gene-specific primers are shown in Table S2 (see online supplementary material).

### Overexpression and mutant plants construction

*MAPK20* and *ATG6* CRISPR/Cas9 vector were constructed as previously described [70]. The target sequence of *MAPK20* (TCAGCCATTGACACGCACAC) and *ATG6* (GGTAAAGTCCGACCCTTATC) were designed using CRISPR-P web tool [71]. The constructed *MAPK20* and *ATG6* knockout plasmids were transformed into *Agrobacterium tumefaciens* strain EHA105 and transformed into tomato seeds as previously described [72]. The *mapk20* and *atg6* lines were mutated at the 3rd base of the PAM and stopped translation immediately (Figs S2 and S8, see online supplementary material). Two independent F_2_ mutant lines of *mapk20* and *atg6* were used in this study, respectively.

To generate the tomato *MAPK20* overexpressing vector, an 1866 bp CDS was amplified and ligated into the pFGC1008-HA vector. The plasmids were transformed into WT to obtain *MAPK20* overexpressing plants. The independent T_2_ lines of *MAPK20* overexpressing plants (OE-*MAPK20*–10 and OE-*MAPK20*–13) were used in this study.

To knockout *ATG6* gene in *MAPK20* overexpressing plants, OE-*MAPK20*–10 plants crossed with *atg6–6* or *atg6–8* mutant plants, respectively. The homozygous F_2_ lines of OE-*MAPK20*–10/*atg6–6* and OE-*MAPK20*–10/*atg6–8* plants were used. The protein level of MAPK20 was analysed by immunoblotting (Fig. S14, see online supplementary material) and the mutation of *ATG6* was confirmed by sequencing (Fig. S8, see online supplementary material).

### Pollen morphology, viability, and germination rate analysis

The pollen morphology was detected with SEM according to the method of Chen *et al.* [19]. The pollen viability and germination *in vitro* were analysed as described by Xie *et al.* [73]. The pollen germination *in vivo* was measured as previously described [11]. PI3P and its carrier were added into the germination medium as described by Xu *et al.* [45] (see Methods S1, see online supplementary material).

### Yeast two hybrid assay

The CDS of *MAPK20* and *ATGs* were amplified with the specific primers (Table S3, see online supplementary material) by PCR. The PCR products were inserted into the pGBKT7 and pGADT7 vectors, respectively. The constructed pGBKT7 and pGADT7 vectors were transformed into a Y2H gold yeast strain according to the method described previously [74].

### Pull-down assay

The CDS of *ATG6* and *MAPK20* was amplified and inserted into pGEX4T-1 or pET32a to generate GST-ATG6 or HIS-MAPK20 recombinant protein, respectively. For pull-down assays, GST-ATG6 protein was first incubated with GST-tag resin (Beyotime, Shanghai, China, P2251) at 4°C for 2 h with slow rotation. Then, HIS-MAPK20 was added and incubated for 2 h. The beads were centrifuged at 4°C,1000 *g* for 2 min, washed five times with pull-down buffer, and boiled with 2 × SDS loading buffer. The proteins were analysed with an anti-GST (Abmart, Shanghai, China, M20007) or anti-HIS (Abmart, Shanghai, China, M30111) antibody.

### Split-luciferase assay

The split-luciferase assay was performed as previously described [75]. The CDS of *ATG6* and *MAPK20* was inserted into the pCAMBIA1300-nLUC and pCAMBIA1300-cLUC, respectively. *A. tumefaciens* strain GV3101 carrying the indicated plasmids infiltrated into the *N. benthamiana* leaves. Luciferase luminescence was detected after 2 d of infiltration.

### Co-IP assay

The CDS of *ATG6* and *MAPK20* was introduced into the pFGC1008-HA and pFGC5941-MYC vector, respectively, and transformed into *A. tumefaciens* strain GV3101. After inoculation into *N. benthamiana* leaves for 2 d, total proteins were extracted with isolation buffer [100 mM HEPES (pH 7.5, Solarbio, Beijing, China, H8090), 5 mM EDTA, 5 mM EGTA (Solarbio, E8050), 10% glycerol, 10 mM Na_3_VO_4_ (Solarbio, G8460), 10 mM NaF, 50 mM β-glycerophosphate (Sigma-Aldrich, St. Louis, MO, USA, G9422), 1 mM PMSF, 1 × protease inhibitor cocktail tablet (MedChemExpress, Monmouth Junction, NJ, USA, HY-K0011), 7.5% polyvinylpolypyrrolidone (Solarbio, P8070)] and were centrifuged at 12000 *g*, 4°C for 15 min. The proteins were immunoprecipitated with anti-HA magnetic beads (MedChemExpress, HY-K0201). ATG6-HA and MYC-MAPK20 were analysed with an anti-HA (Abmart, M20003) and anti-MYC antibody (Abmart, M20002), respectively.

### BiFC assay and ATG8f co-localization

The *MAPK20* CDS was inserted into pFGC5941-N-YFP, and *ATG6* was ligated into pFGC5941-C-YFP vector, respectively. The DNA fragment of mCherry-ATG8f was obtained by PCR using specific primers and the PCR product was inserted into the pFGC1008 vector. The primers used for BiFC assays and ATG8f co-localization were listed in Table S3 (see online supplementary material). The inoculation of *N. benthamiana* was carried out as previously described [76].

### LC–MS/MS analysis and ATG6 phosphorylation assays

To generate the constitutively active MKK2, T215D/S221D double mutant was amplified and introduced into the pFGC1008-Flag vector. The *A. tumefaciens* GV3101 harboring plasmid with ATG6-HA (pFGC1008-HA), MYC-MAPK20 (pFGC5941-MYC), and MKK2^DD^-Flag (pFGC1008-Flag) mixed to infiltrate the *N. benthamiana* leaves. Leaves injected with GV3101 only carrying ATG6-HA or a mixture of ATG6-HA and MYC-MAPK20 were used as control. After injection for 2 d, total proteins were extracted with Co-IP extraction buffer and were immunoprecipitated with anti-HA magnetic beads. Then, the proteins were separated with 12% SDS-PAGE gel and stained with Coomassie brilliant blue R-250 (Solarbio, C8430). The target bands were sent to Shanghai Applied Protein Technology Co., Ltd for LC–MS/MS analysis.

To further analyse the phosphorylation of MAPK20 to ATG6 *in vitro*, the serine at position 265 of ATG6 protein was mutant to alanine (ATG6^S265A^) and inserted into the pGEX4T-1 vector. The CDS of *MKK2^DD^* and *MAPK20* was amplified with the specific primers (Table S3, see online supplementary material) and ligated into the pET32a and pMAL-C2 vector, respectively. The recombinant proteins of HIS-MKK2^DD^, MBP-MAPK20, GST-ATG6, and GST-ATG6^S265A^ were obtained as above described in pull-down assay. The phosphorylation assays were performed as previously described with modification [77]. MBP-MAPK20 (7.5 μg) was activated by 0.25 μg HIS-MKK^2DD^ in 50 μL of reaction buffer (10 mM MgCl_2_, 20 mM HEPES pH 7.5, 1 mM DTT) containing 0.2 mM ATP for 1.5 h at 25°C. Then, the activated MAPK20 was used to phosphorylate GST-ATG6 or GST-ATG6^S265A^ at the ratio of 1:10 in the same reaction buffer containing 1 mM ATP for 2 h at 30°C. CIAP (Invitrogen, Carlsbad, CA, USA, 18 009) was used to confirm the phosphorylation according to the instructions.

To further analyse the phosphorylation of ATG6 by MAPK20 *in vivo*, we constructed pFGC5941-ATG6-GFP and pFGC5941-ATG6^S265A^-GFP vectors. The *A. tumefaciens* containing the indicated binary vector was infiltrated into *N*. *benthamiana* leaves for 2 d, then the protein was extracted and immunoprecipitated with GFP agarose beads (Chromotek, Planegg-Martinsried, Germany, AB2827596). The phosphorylation of ATG6 protein *in vitro* and *vivo* was detected with an anti-phosphoserine (anti-pSer) antibody (SantaCruz, Dallas, TX, USA, sc-81 514).

### MDC staining and immunoblotting analysis of ATG8 protein level

MDC staining was performed as the method described by Xie *et al.* [73]. For detecting the level of ATG8, the proteins were isolated and immunoblotted as described in a previous study [78].

### Semi-thin section and PI3P content measurement

Semi-thin section observation was performed following the method described by Yan *et al.* [11] (see Methods S2, see online supplementary material). PI3P content was measured according to the instructions of a PI(3)P Mass ELISA Kit (Echelon Biosciences, Salt Lake, UT, USA, K-3300, see Methods S3, see online supplementary material).

### Statistical analysis

Each determination was performed in at least three independent replicates. SPSS 18.0 software was used for statistical analysis. Different letters between treatments indicate significant differences at *P* < 0.05 with Tukey’s test.

## Acknowledgements

We are grateful for Prof. Gang Lu of Zhejiang University for donating the CRISPR/Cas9 vector, and we thank Dr Ze Wu of Nanjing Agricultural University for donating the split-luciferase vector. This work was supported by the National Key R&D Program of China (2023YFD2300700), the National Natural Science Foundation of China (32272790), Zhejiang Province Science and Technology Plan (2023C02001), the Starry Night Science Fund of Zhejiang University Shanghai Institute for Advanced Study (SN-ZJU-SIAS-0011), Collaborative Promotion Program of Zhejiang Provincial Agricultural Technology of China (2023ZDXT05), and the Jiangsu Provincial Association for Science and Technology Youth Science and Technology Talent Support Project (TJ-2023-003).

## Author contributions

Y.W., J.Y., and J.Z conceived and designed the experiments. Y.W., D.X., X.Z., M.G., Z.Q., and P.Y. performed the experiments. Y.W., D.X., and J.Z. analysed the data. Y.W., D.X., and J.Z. wrote the article and all authors revised and approved the final manuscript.

## Data availability

The data supporting the findings of this study are available within the paper and its supplementary information files.

## Conflict of interest statement

The authors declare no potential conflicts of interest.

## Supplementary data

Supplementary data is available at *Horticulture Research* online.

## Supplementary Material

Web_Material_uhae069
