# Supplementary material for: MAPK20-mediated ATG6 phosphorylation is critical for pollen development in Solanum lycopersicum L
Source: Hortic Res. 2024 Mar 6;11(5):uhae069. doi: 10.1093/hr/uhae069 (PMC11079483; doi:10.1093/hr/uhae069)
Supplement: Web_Material_uhae069 [file web_material_uhae069.zip › Supplemental Figures-20240203.pdf]

Figure S1

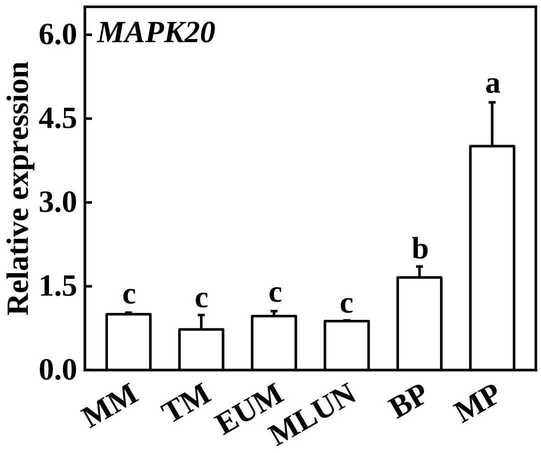

Figure S1. Analysis of *MAPK20* expression profile in tomato stamen at different stages of pollen development using qPCR. BP, binucleate pollen stage; EUM, early uninucleate microspore stage; MLUN, middle and later period of uninucleate microspore stage; MM, microspore mother stage; MP, mature pollen stage; TM, tetrad stage.

Figure S2

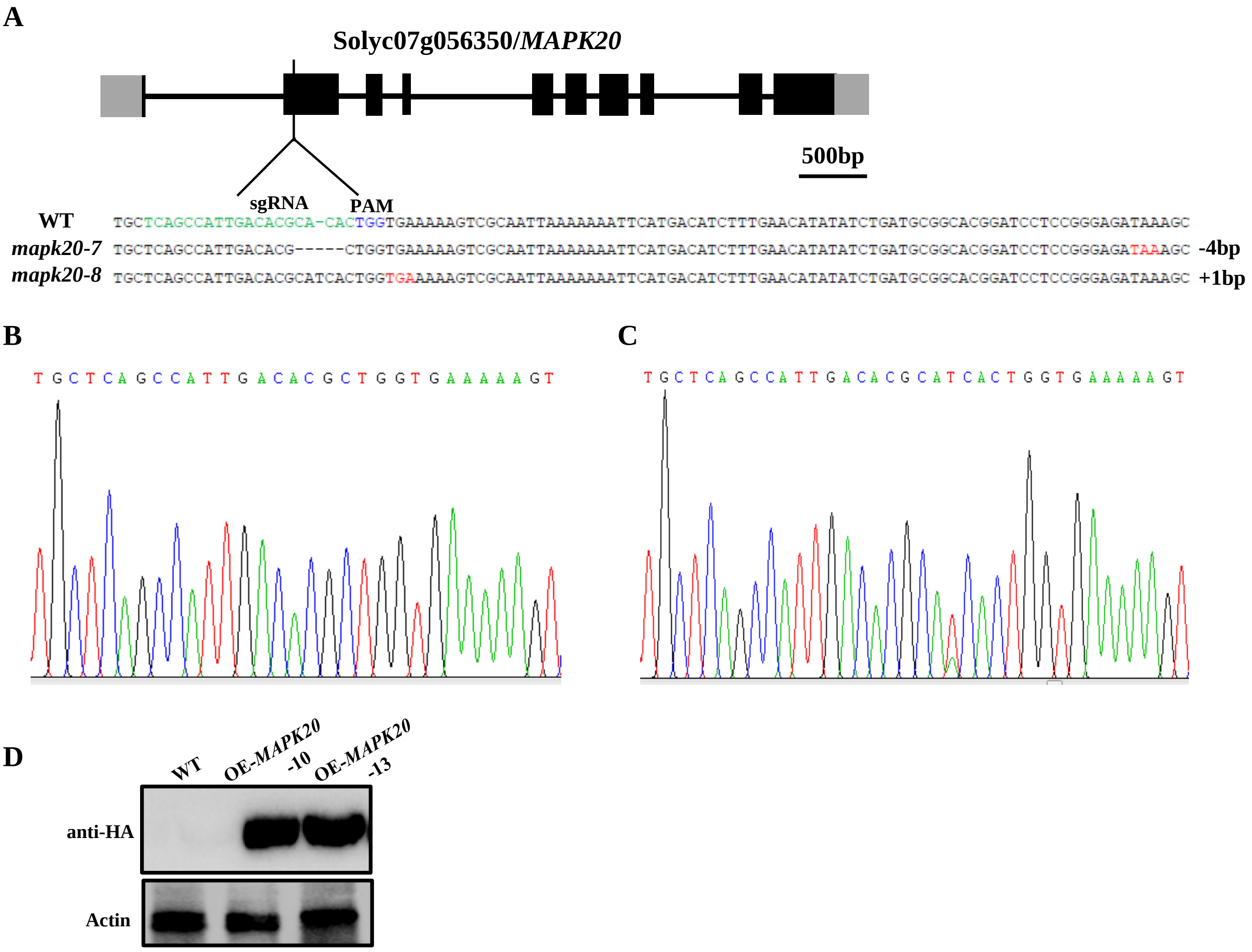

Figure S2. Identification of *MAPK20* knockout mutants and overexpressing plants. **A** DNA sequence comparison of WT, *mapk20-7*, and *mapk20-8*. Sequencing analysis showed that *mapk20-7* mutant contained the 4 bp deletion in the second extron, and *mapk20-8* mutant contained a 1 bp insertion in the second extron and stopped translation immediately. The protospacer adjacent motif (PAM) is indicated by blue. Single guide RNA (sgRNA) is indicated by green. Stop codon is indicated by red. **B** Sequencing result of *mapk20-7* mutant. **C** Sequencing result of *mapk20-8* mutant. **D** Immunoblotting analysis of *MAPK20* overexpressing plants. Total proteins were extracted from the leaves and equal amounts of proteins were subjected to SDS-PAGE, and probed with an anti-HA monoclonal antibody. Actin was used as a loading control for the immunoblotting analysis.

Figure S3

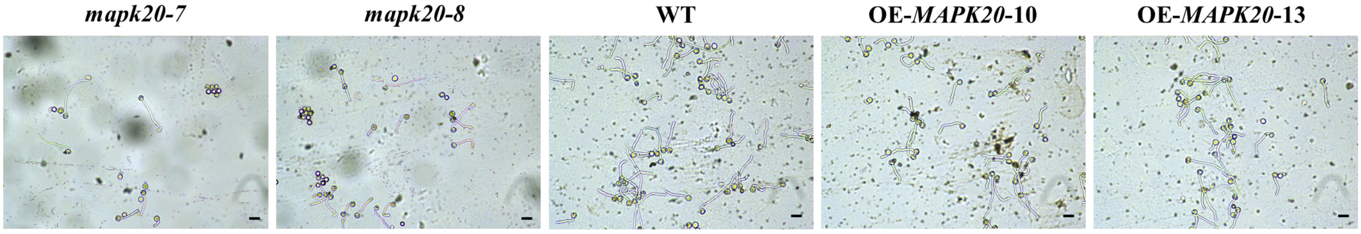

Figure S3. Pollen germination of WT, *MAPK20* gene knockout and overexpressing plants. The mature pollen grains were scattered in the germination medium. The pollen tube grew at 28°C for 1 h in the dark and the germinated pollen grains were measured by a fluorescence microscope. OE, overexpressing; WT, wild-type. Bars: 50 μm.

Figure S4

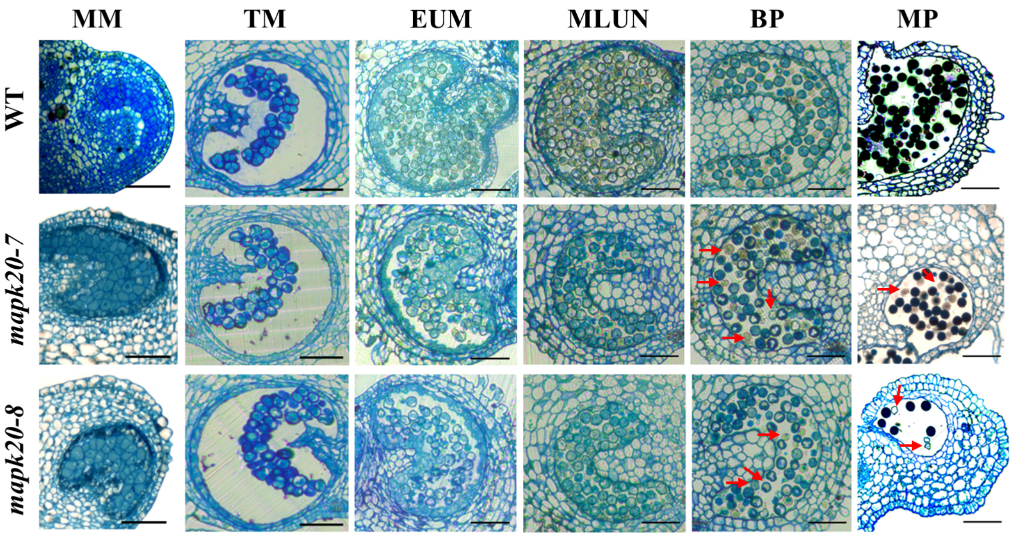

Figure S4. Semi-thin section comparison of anther and pollen development between WT and *mapk20* plants. Red arrow indicates the aborted pollen grain. Bars: 100 μm. BP, binucleate pollen stage; EUM, early uninucleate microspore stage; MLUN, middle and later period of uninucleate microspore stage; MM, microspore mother stage; MP, mature pollen stage; WT, wild-type; TM, tetrad stage.

Figure S5

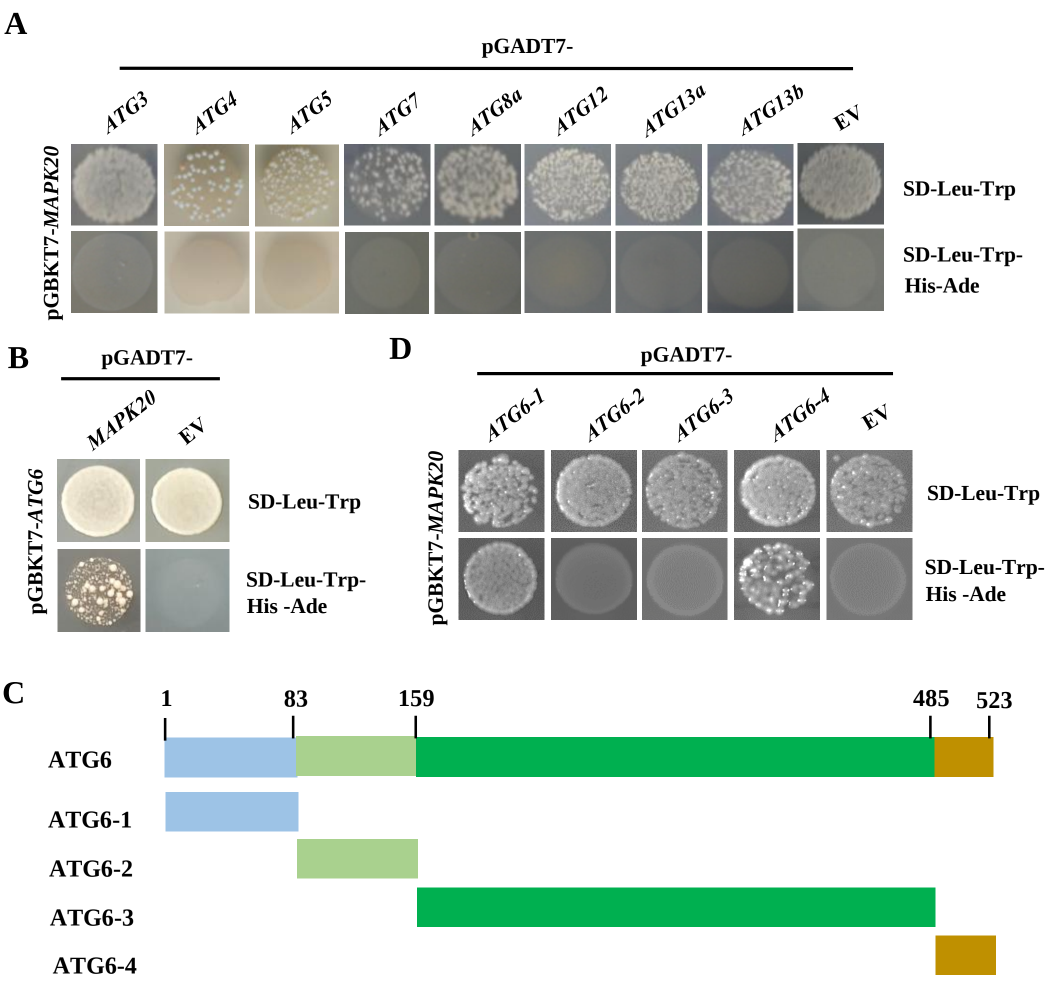

Figure S5. Yeast two-hybrid assays of detecting the interaction between MAPK20 and ATG proteins. **A** Other ATGs did not interacted with MAPK20. **B** Yeast two-hybrid assays analysis MAPK20 interaction with ATG6 proteins. **C** and **D** Four truncations (ATG6-1 to ATG6-4) of ATG6 were fused to the prey vector (pGADT7-*ATGs*) and co-transformed into yeast with pGBKT7-*MAPK20*, and found that MAPK20 interacted with ATG6-1 (1-83 aa) and ATG6-4 (485-523 aa). The empty vector (EV) of pGADT7 was used as negative control.

Figure S6

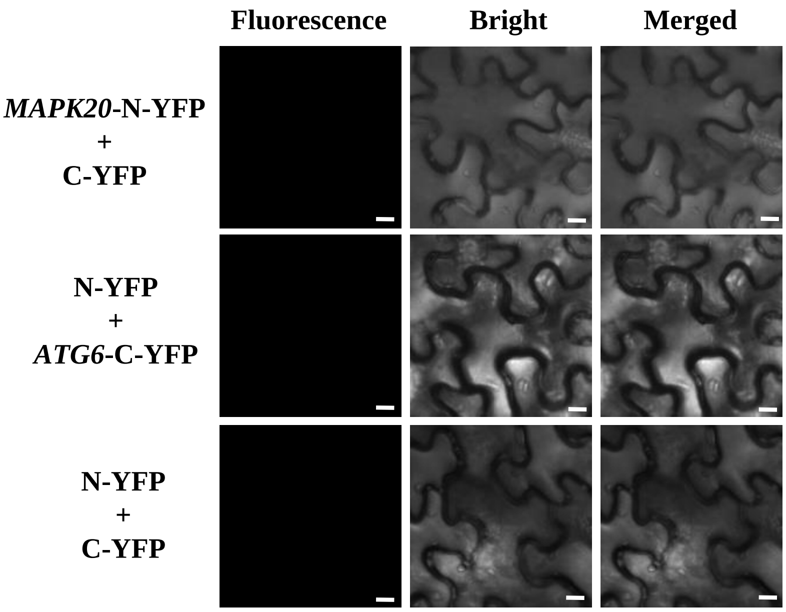

Figure S6. Bimolecular fluorescence complementation analysis of MAPK20 interaction with ATG6. No fluorescence was observed when *MAPK20*-N-YFP was co-expressed with unfused C-YFP or when unfused N-YFP was co-expressed with *ATG6*-C-YFP or when unfused N-YFP was co-expressed with unfused C-YFP. Bars: 25 μm.

Figure S7

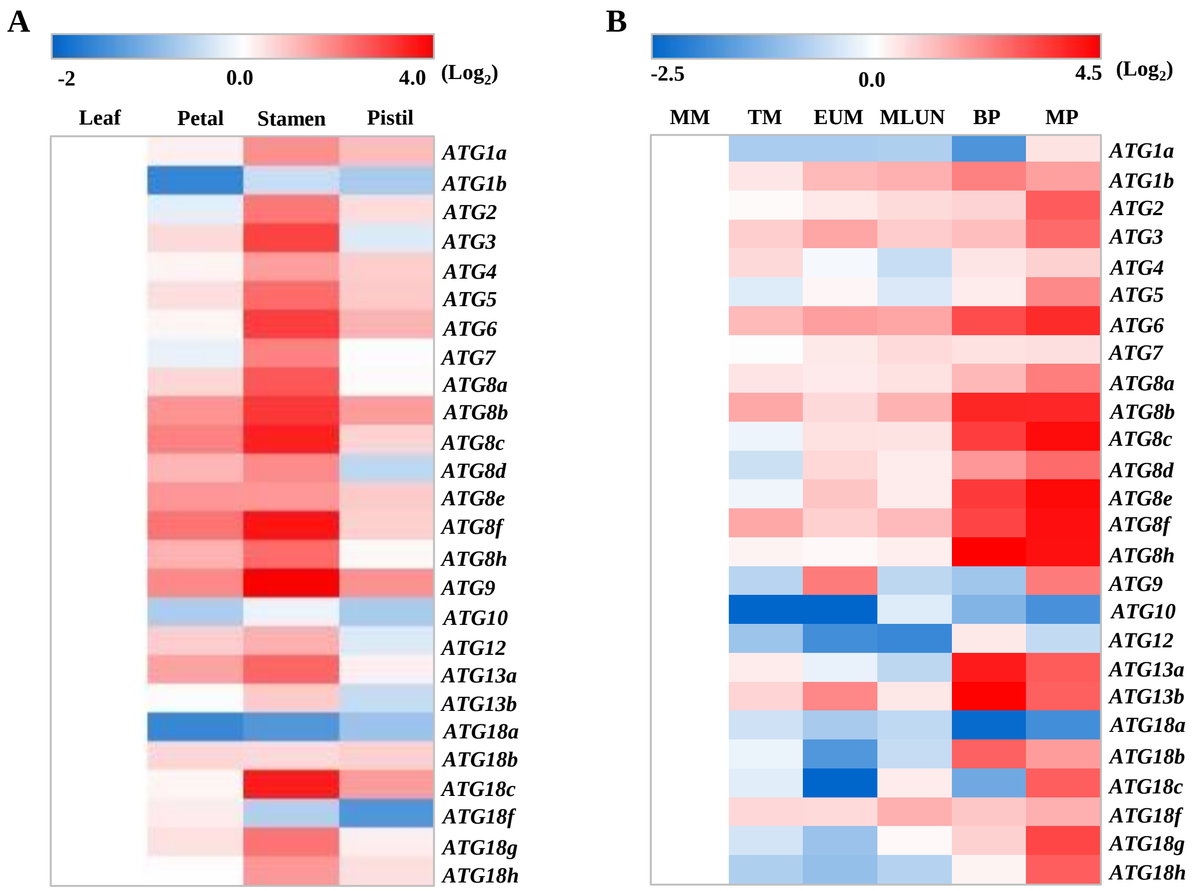

Figure S7. Analysis of *ATG* genes expression profiles in tomato using qPCR. **A** Heat-map showing the expression profiles of *ATG* genes in tomato leaf and floral tissues (petal, stamen, and pistil). **B** Heat-map showing the expression profiles of *ATG* genes in stamens at different stages of pollen development. The color bar at the top shows levels of expression. Transcript levels were determined using qPCR and the cluster analysis was performed using MeV version 4.9. BP, binucleate pollen stage; EUM, early uninucleate microspore stage; MLUN, middle and later period of uninucleate microspore stage; MM, microspore mother stage; MP, mature pollen stage; TM, tetrad stage.

Figure S8

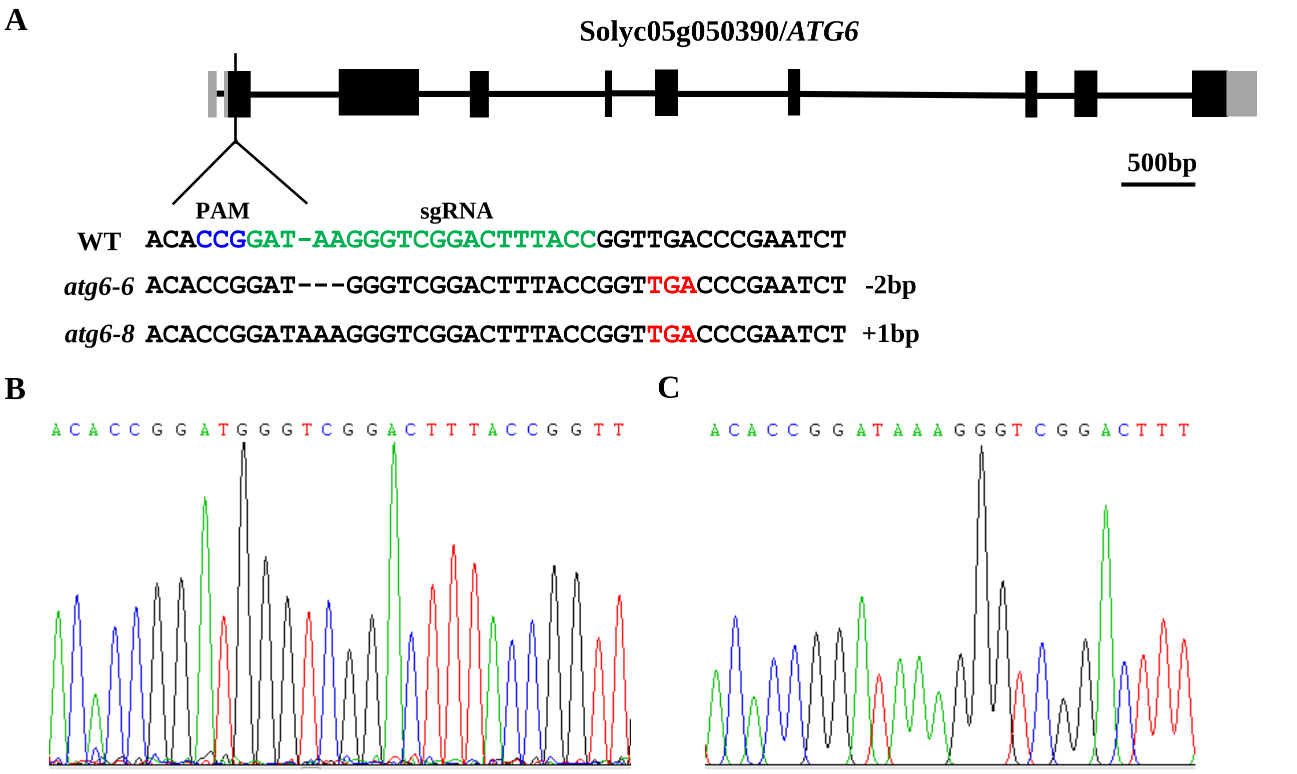

Figure S8. Identification of *atg6* mutants. **A** DNA sequence comparison of WT, *atg6-6*, and *atg6-8*. Sequencing analysis showed that *atg6-6* mutant contained the 2 bp deletion in the second extron and *atg6-8* mutant contained a 1 bp insertion in the second extron and stopped translation immediately. The protospacer adjacent motif (PAM) is indicated by blue. Single guide RNA (sgRNA) is indicated by green. Stop codon is indicated by red. **B** Sequencing result of *atg6-6* mutant. **C** Sequencing result of *atg6-8* mutant.

Figure S9

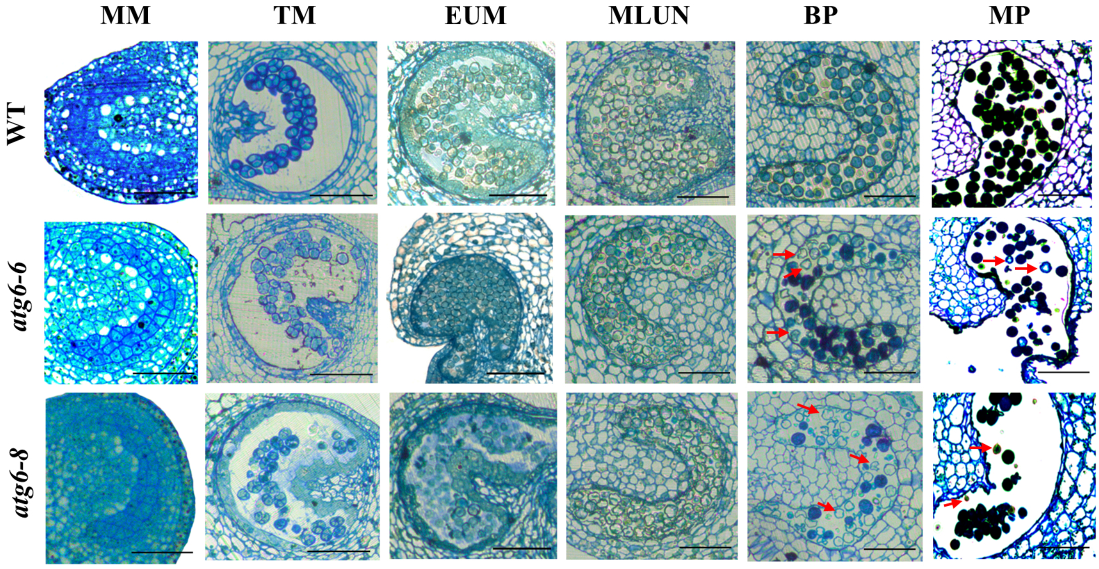

Figure S9. Semi-thin section comparison of anther and pollen development between WT and *atg6* plants. Red arrow indicates the aborted pollen grain. Bars: 100 µm. BP, binucleate pollen stage; EUM, early uninucleate microspore stage; MLUN, middle and later period of uninucleate microspore stage; MM, microspore mother stage; MP, mature pollen stage; WT, wild-type; TM, tetrad stage.

Figure S10

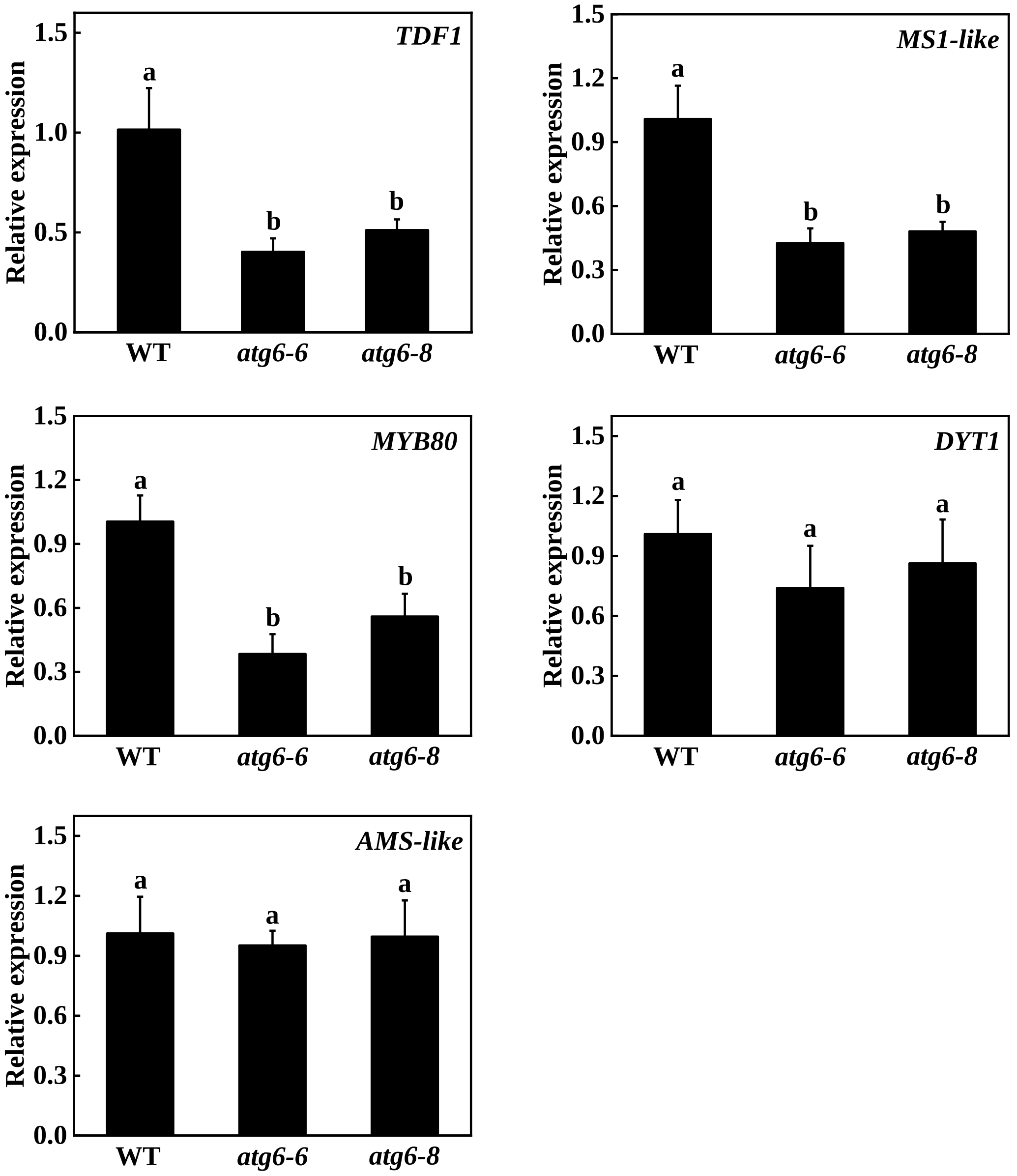

Figure S10. *ATG6* regulates the expression of pollen development-related genes. Results represent the means ± SD. Means with different letter showed significantly differ ($P < 0.05$).

Figure S11

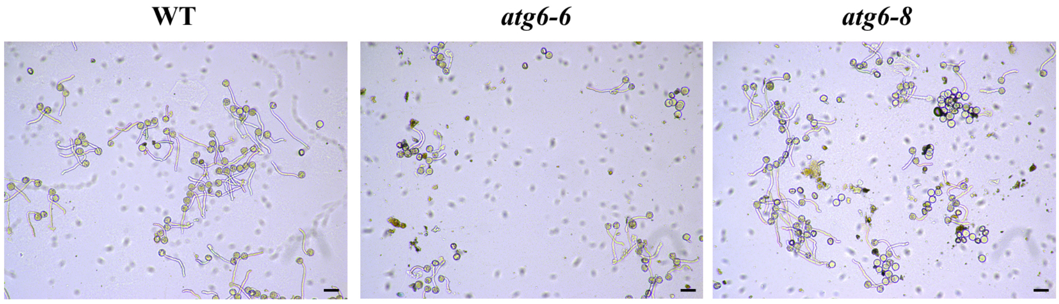

Figure S11. Pollen germination of WT and *ATG6* gene knockout plants. The mature pollen grains were scattered in the germination medium. The pollen tube grew at 28°C for 1 h in the dark and the germinated pollen grains were measured by a fluorescence microscope. WT, wild-type. Bars: 50 μm.

Figure S12

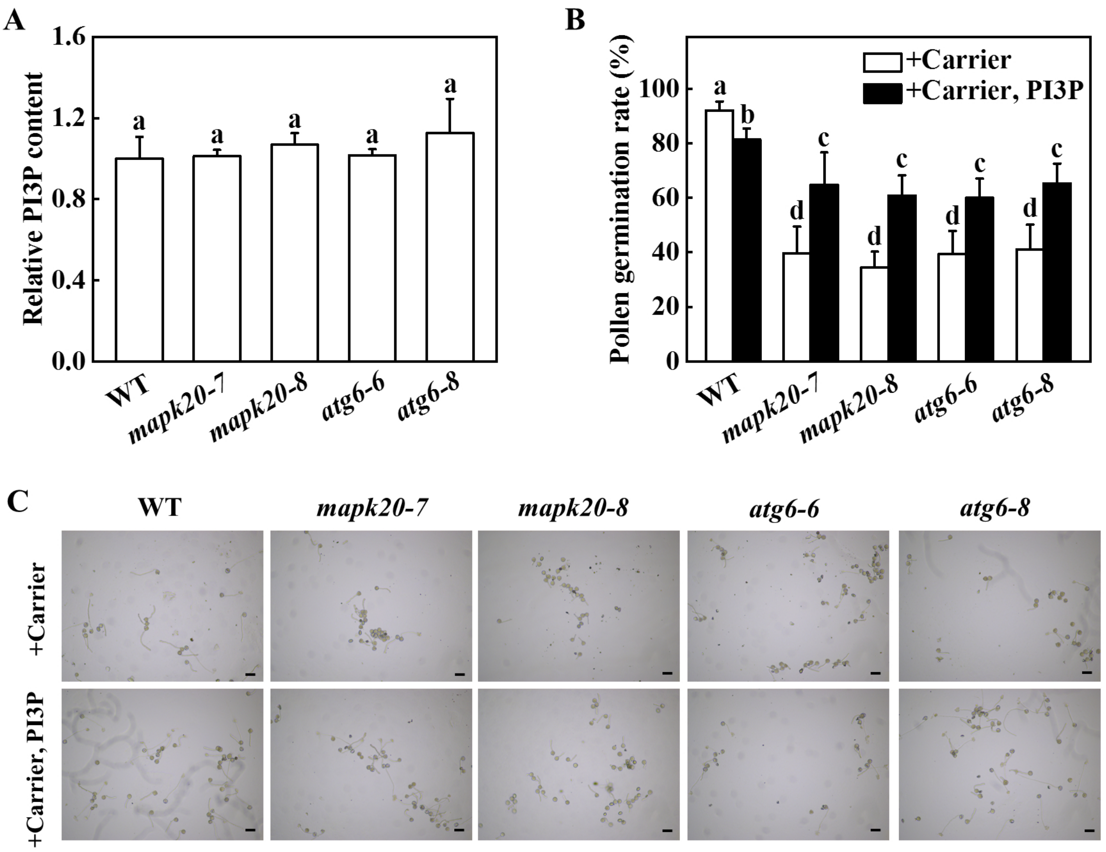

Figure S12. Functional analysis of PI3P on pollen germination. **A** PI3P content in the leaves of WT, *mapk20*, and *atg6* plants. The leaves were collected from six-week-old plants, and PI3P content was measured using a PI(3)P Mass ELISA Kit. The relative PI3P content was calculated relative to WT, which was set to 1. **B and C** Effects of exogenous PI3P on the germination of WT, *mapk20*, and *atg6* pollen grains. Bars: 50 μm. Results represent the means ± SD. Means with different letter showed significantly differ ($P < 0.05$).

Figure S13

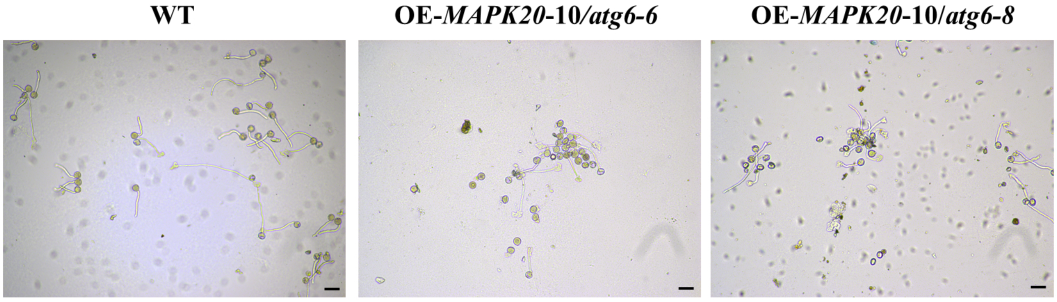

Figure S13. Pollen germination of WT and OE-*MAPK20/atg6* plants. The mature pollen grains were scattered in the germination medium. The pollen tube grew at 28°C for 1 h in the dark and the germinated pollen grains were measured by a fluorescence microscope. OE, overexpressing; WT, wild-type. Bars: 50 μm.

Figure S14

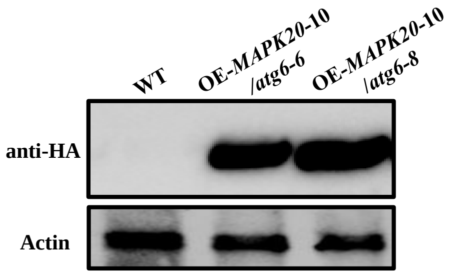

Figure S14. The protein level of MAPK20 in OE-*MAPK20*/*atg6* plants. Total proteins were extracted from the leaves and equal amounts of proteins were subjected to SDS-PAGE, and probed with an anti-HA monoclonal antibody. Actin was used as a loading control for the immunoblotting analysis.
