# Supplementary material for: MAPK20-mediated ATG6 phosphorylation is critical for pollen development in Solanum lycopersicum L
Source: Hortic Res. 2024 Mar 6;11(5):uhae069. doi: 10.1093/hr/uhae069 (PMC11079483; doi:10.1093/hr/uhae069)
Supplement: Web_Material_uhae069 [file web_material_uhae069.zip › Supplemental Methods.pdf]

Methods S1.

Exogenous PI3P on pollen germination

To analyze the effect of PI3P on pollen germination, PI3P (Echelon, P-3016) and it carrier (Carrier 3, Echelon, P-9C3) were added into the pollen germination medium (120 g L^{-1} sucrose, 0.05 g L^{-1} H_3BO_3, 0.3 g L^{-1} $Ca(NO_3)_2 \cdot 4H_2O$, 0.2 g L^{-1} $MgSO_4 \cdot 7H_2O$, and 0.1 g L^{-1} KNO_3, 0.1% agarose, pH 6.5). PI3P and carrier was mixed at the ratio of 1:1 to form the PI3P-carrier complex, and then added into the germination medium at the final concentration of 6.25×10^{-3} mg mL^{-1}. The mature pollen grains were scattered in the germination medium with PI3P and carrier or only carrier. The pollen tube grew at 28°C for 1 h in the dark and the germinated pollen grains were measured by fluorescence microscope (Leica, Germany).

Methods S2.

Semi-thin section analysis of anther

Anthers of different developmental stage were fixed with 2.5% glutaraldehyde in 0.1 M PBS buffer (pH 7.0) at 4°C for 12 h in the dark. Then, washed with PBS buffer for three times, and again fixed in 1% (v/v) osmium tetroxide at room temperature for 2 h; then the samples were dehydrated in a graded ethanol series (30 to 100%; v/v) and embedded in Epon 812. For semi-thin section observation, the samples were sliced with LKB11800 semi-thin slicer (Leica, Germany) to obtain 2 μm thickness of sections and stained with 1% methylene blue. The semi-thin sections were observed by microscope (Leica, Germany).

Methods S3.

PI3P content measurement

Tomato leaves (0.35 g) were ground in liquid nitrogen, transferred into a 10 mL tubes, and added 3 mL 5% TCA/1 mM EDTA. After vortex for 30 s, the tubes were centrifuged at 3000 r for 5 min, discarded the supernatant, and repeat washed one time. The acidic lipids were extracted by adding 2.25 mL $CHCl_3$, CH_3OH, and HCl (80:40:1, v:v) and vortex for 25 min at room temperature. The tube was centrifuged at 3000 r for 5 min,

transferred the supernatant to a new 10 mL centrifuge tube, and added 0.75 mL of $CHCl_3$ and 1.35 mL of 0.1 N HCl. After vortex for 30 s, the tubes were centrifuged at 3000 r for 5 min, the lowest organic phase was collected into a new 2 mL tube, and dried in a vacuum dryer. Then, PI3P content was detected using a PI(3)P Mass ELISA Kit (Echelon Biosciences, K-3300) according its instructions. Briefly, the dried lipids were rehydrated by adding 200 μL of PBST 3% Protein Stabilizer (PBS-T 3%PS), sonicated for 10 min in a room temperature water bath, and vortex for 2 min. Then, the rehydrated samples were diluted for 10 fold with PBS-T 3%PS, and incubated with a PI3P detector for 1 h at room temperature. Subsequently, the incubated samples were added to a PI3P coated microplate for competitive binding for 1 h at room temperature, and the secondary detector was added into the plate to incubate for another 1 h. The plate was added 100 μL of TMB solution (K-TMB1) to each well, incubated in the dark for 30 min, and 50 μL of 1 N H_2SO_4 was added to stop the reaction. The absorbance was read at 450 nm on a FlexA-200 Microplate reader (Hangzhou Aosheng Instrument Co., Ltd, Hangzhou, China). The content of PI3P was calculated according to standard curve.
