# Supplementary material for: MAPK20-mediated ATG6 phosphorylation is critical for pollen development in Solanum lycopersicum L
Source: Hortic Res. 2024 Mar 6;11(5):uhae069. doi: 10.1093/hr/uhae069 (PMC11079483; doi:10.1093/hr/uhae069)
Supplement: Web_Material_uhae069 [file web_material_uhae069.zip › Supplemental Tables-20240203.pdf]

Table S1. Liquid chromatography-tandem mass spectrometry analysis of ATG6 phosphorylation site.

**FileScan**	**Sequence**	**MH+**	**Diff(MH+)**	**Score**	**ExpectValue**	**Modification**
MKK2_MAPK20_ATG6,2689	K.DRENNIRPDK.C	1256.63415	0.0002	20.34	4.62E-04	
MKK2_MAPK20_ATG6,14881	K.EFSEFANSK.D	1058.4789	0.00019	33.79	2.09E-05	
MKK2_MAPK20_ATG6,15175	K.EFSEFANSK.D	1058.4789	-0.00105	37.36	9.18E-06	
MKK2_MAPK20_ATG6,2391	K.ELEM&KS^GR.F	1045.43835	-0.00097	23.54	2.21E-04	15.994919 Oxidation (M); 79.966324 Phospho (ST)
MKK2_MAPK20_ATG6,3880	K.EVEDVNR.D	860.41083	0.0004	25.76	1.33E-04	
MKK2_MAPK20_ATG6,4044	K.EVEDVNR.D	860.41083	0.00062	25.76	1.33E-04	
MKK2_MAPK20_ATG6,18638	K.FFPDSSYR.S	1018.46287	-0.00035	46.8	1.04E-06	
MKK2_MAPK20_ATG6,18933	K.FFPDSSYR.S	1018.46287	-0.00047	42.71	2.68E-06	
MKK2_MAPK20_ATG6,19507	K.FFPDSSYR.S	1018.46287	0.00207	27.48	8.93E-05	
MKK2_MAPK20_ATG6,1673	K.GSSATPDK.G	762.36282	0.00014	66.72	1.06E-08	
MKK2_MAPK20_ATG6,1838	K.GSSATPDK.G	762.36282	0.0001	46.05	1.24E-06	
MKK2_MAPK20_ATG6,2947	K.IEEEER.K	804.37335	0.00077	32.46	5.68E-05	
MKK2_MAPK20_ATG6,3173	K.IEEEER.K	804.37335	-0.00057	28.54	1.40E-04	
MKK2_MAPK20_ATG6,2085	K.IEEEERK.L	932.46831	-0.00042	26.94	1.01E-04	
MKK2_MAPK20_ATG6,4	K.IENDK.V	618.30931	0.00012	31.43	3.60E-05	
MKK2_MAPK20_ATG6,123	K.IENDK.V	618.30931	0.00018	28.71	6.73E-05	
MKK2_MAPK20_ATG6,241	K.IENDK.V	618.30931	0.00024	27.78	8.34E-05	
MKK2_MAPK20_ATG6,359	K.IENDK.V	618.30931	0.00018	30.83	4.13E-05	
MKK2_MAPK20_ATG6,477	K.IENDK.V	618.30931	0.00016	28.96	6.35E-05	
MKK2_MAPK20_ATG6,593	K.IENDK.V	618.30931	0.0002	29.06	6.21E-05	
MKK2_MAPK20_ATG6,711	K.IENDK.V	618.30931	0.0001	26.3	1.17E-04	
MKK2_MAPK20_ATG6,828	K.IENDK.V	618.30931	0.00018	20.93	4.04E-04	
MKK2_MAPK20_ATG6,945	K.IENDK.V	618.30931	0.00018	23.55	2.21E-04	
MKK2_MAPK20_ATG6,20175	K.IENDKVESYSITQSFNK.Q	2001.97635	-0.00094	75.73	2.67E-09	
MKK2_MAPK20_ATG6,13494	K.IIPM&GSYPR.I	1049.54481	-0.00125	35.64	1.36E-05	15.994919 Oxidation (M)
MKK2_MAPK20_ATG6,13744	K.IIPM&GSYPR.I	1049.54481	0.00038	30.86	4.10E-05	15.994919 Oxidation (M)
MKK2_MAPK20_ATG6,14016	K.IIPM&GSYPR.I	1049.54481	-0.00113	35.78	1.32E-05	15.994919 Oxidation (M)

MKK2_MAPK20_ATG6,18848	K.IIPM&GSYPR.I	1049.54481	0.00049	22.27	2.96E-04	15.994919 Oxidation (M)
MKK2_MAPK20_ATG6,19151	K.IIPMGSYPR.I	1033.54989	0.00033	33.07	2.47E-05	
MKK2_MAPK20_ATG6,7174	K.IIPMGSYPR.I	1033.54989	0.00065	25.43	1.43E-04	
MKK2_MAPK20_ATG6,8036	K.LDKEVEDVNR.D	1216.61679	-0.00025	46.95	1.01E-06	
MKK2_MAPK20_ATG6,12055	K.LDKEVEDVNR.D	1216.61679	-0.00054	23.75	2.11E-04	
MKK2_MAPK20_ATG6,8249	K.LETAIEETEK.Q	1162.58374	0.00015	56.78	3.15E-07	
MKK2_MAPK20_ATG6,8354	K.LKIEEEER.K	1045.55237	0.00382	22.65	2.72E-04	
MKK2_MAPK20_ATG6,8499	K.LKIEEEER.K	1045.55237	-0.00044	56.58	1.10E-07	
MKK2_MAPK20_ATG6,5658	K.LKIEEEER.K	1045.55237	0.00097	41.83	3.28E-06	
MKK2_MAPK20_ATG6,15104	K.LKIEEEERK.L	1173.64732	0.00024	27.37	9.16E-05	
MKK2_MAPK20_ATG6,15379	K.QCAVVTAELK.E	1118.58742	-0.00055	22.63	2.73E-04	
MKK2_MAPK20_ATG6,6186	K.QCAVVTAELK.E	1118.58742	0.00169	48.03	7.87E-07	
MKK2_MAPK20_ATG6,6436	K.QENWTK.A	805.38388	-0.00025	22.47	2.83E-04	
MKK2_MAPK20_ATG6,3	K.QENWTK.A	805.38388	-0.00051	25.69	1.35E-04	
MKK2_MAPK20_ATG6,124	K.QPTNTK.F	688.36242	0.00014	44.1	1.95E-06	
MKK2_MAPK20_ATG6,19385	K.QPTNTK.F	688.36242	0.0001	48.09	7.76E-07	
MKK2_MAPK20_ATG6,19389	K.TEVSQAHLELLK.R	1367.75288	-0.00062	57.07	9.82E-08	
MKK2_MAPK20_ATG6,19657	K.TEVSQAHLELLK.R	1367.75288	0.00076	75.79	1.32E-09	
MKK2_MAPK20_ATG6,16147	K.TEVSQAHLELLK.R	1367.75288	-0.00008	42.22	3.00E-06	
MKK2_MAPK20_ATG6,20376	K.TEVSQAHLELLKR.T	1523.85399	-0.00148	30.13	4.85E-05	
MKK2_MAPK20_ATG6,24474	K.VESYSITQSFNK.Q	1402.68487	0.00055	24.55	1.75E-04	
MKK2_MAPK20_ATG6,24513	R.AFDIATTQTQIEQPLCLECM&R.V	2541.17821	-0.00054	43.18	2.40E-06	15.994919 Oxidation (M)
MKK2_MAPK20_ATG6,24596	R.AM&EESFVVLPPPAASVYK.C	1950.98811	-0.00149	63.47	2.25E-08	15.994919 Oxidation (M)
MKK2_MAPK20_ATG6,24746	R.AM&EESFVVLPPPAASVYK.C	1950.98811	0.00047	39.68	5.38E-06	15.994919 Oxidation (M)
MKK2_MAPK20_ATG6,24762	R.AMEESFVVLPPPAASVYK.C	1934.99319	0.00263	36.42	1.14E-05	
MKK2_MAPK20_ATG6,7775	R.AMEESFVVLPPPAASVYK.C	1934.99319	-0.00064	44.71	1.69E-06	
MKK2_MAPK20_ATG6,8004	R.DAILAK.T	630.38208	-0.00003	36.62	1.09E-05	
MKK2_MAPK20_ATG6,2502	R.DAILAK.T	630.38208	0.00013	32.43	2.86E-05	
MKK2_MAPK20_ATG6,2517	R.ENNIRPDK.C	985.5061	0.00078	48.25	7.48E-07	

MKK2_MAPK20_ATG6,6719	R.ENNIRPDK.C	985.5061	0.00006	21.76	3.33E-04	
MKK2_MAPK20_ATG6,8100	R.FKELEER.Y	950.49414	0.00029	28.7	6.74E-05	
MKK2_MAPK20_ATG6,8118	R.FKELEER.Y	950.49414	-0.00259	38.98	6.32E-06	
MKK2_MAPK20_ATG6,8360	R.FKELEER.Y	950.49414	-0.0019	25.82	1.31E-04	
MKK2_MAPK20_ATG6,10333	R.FKELEER.Y	950.49414	0.00119	21.95	3.19E-04	
MKK2_MAPK20_ATG6,10455	R.GSAQPDASQFGR.A	1220.56544	-0.00038	48.17	2.29E-06	
MKK2_MAPK20_ATG6,10711	R.GSAQPDASQFGR.A	1220.56544	-0.00028	54.14	5.78E-07	
MKK2_MAPK20_ATG6,15525	R.GSAQPDASQFGR.A	1220.56544	0.00108	25.72	4.02E-04	
MKK2_MAPK20_ATG6,10895	R.GSAQPDASQFGR.A	1220.56544	-0.00482	22.87	7.75E-04	
MKK2_MAPK20_ATG6,10905	R.KLETAIEETEK.Q	1290.67869	0.00077	33.26	2.36E-05	
MKK2_MAPK20_ATG6,11140	R.KLETAIEETEK.Q	1290.67869	-0.0024	37.7	8.49E-06	
MKK2_MAPK20_ATG6,11145	R.KLETAIEETEK.Q	1290.67869	0.00497	47.44	9.02E-07	
MKK2_MAPK20_ATG6,17558	R.KLETAIEETEK.Q	1290.67869	-0.00166	29.44	5.69E-05	
MKK2_MAPK20_ATG6,17835	R.M&ENSYVM&LPK.Q	1243.5697	0.00086	26.08	1.23E-04	15.994919 Oxidation (M)
MKK2_MAPK20_ATG6,17888	R.M&ENSYVM&LPK.Q	1243.5697	0.00102	49.95	5.06E-07	15.994919 Oxidation (M)
MKK2_MAPK20_ATG6,18121	R.M&ENSYVM&LPK.Q	1243.5697	-0.00048	22.47	2.83E-04	15.994919 Oxidation (M)
MKK2_MAPK20_ATG6,20277	R.M&ENSYVM&LPK.Q	1243.5697	0.00074	70.36	4.60E-09	15.994919 Oxidation (M)
MKK2_MAPK20_ATG6,19971	R.M&ENSYVMLPK.Q	1227.57478	-0.00002	57.01	9.95E-08	15.994919 Oxidation (M)
MKK2_MAPK20_ATG6,22349	R.MENSYVM&LPK.Q	1227.57478	0.00098	52.01	3.15E-07	15.994919 Oxidation (M)
MKK2_MAPK20_ATG6,4540	R.MENSYVMLPK.Q	1211.57986	0.00004	22.2	3.01E-04	
MKK2_MAPK20_ATG6,4776	R.NQGSGIPPR.G	925.485	0.00025	36.12	1.22E-05	
MKK2_MAPK20_ATG6,4989	R.NQGSGIPPR.G	925.485	0.00027	64.96	1.60E-08	
MKK2_MAPK20_ATG6,5220	R.NQGSGIPPR.G	925.485	-0.00015	44.76	1.67E-06	
MKK2_MAPK20_ATG6,5457	R.NQGSGIPPR.G	925.485	-0.00005	43.57	2.20E-06	
MKK2_MAPK20_ATG6,5680	R.NQGSGIPPR.G	925.485	-0.00013	41.56	3.49E-06	
MKK2_MAPK20_ATG6,5916	R.NQGSGIPPR.G	925.485	0.00029	47.33	9.25E-07	
MKK2_MAPK20_ATG6,6151	R.NQGSGIPPR.G	925.485	0.00021	45.32	1.47E-06	
MKK2_MAPK20_ATG6,6411	R.NQGSGIPPR.G	925.485	0.00017	44.62	1.73E-06	
MKK2_MAPK20_ATG6,6674	R.NQGSGIPPR.G	925.485	0.00021	42.25	2.98E-06	

MKK2_MAPK20_ATG6,7117	R.NQGSGIPPR.G	925.485	-0.00007	31.66	3.41E-05	
MKK2_MAPK20_ATG6,7360	R.NQGSGIPPR.G	925.485	-0.00005	42.91	2.56E-06	
MKK2_MAPK20_ATG6,7622	R.NQGSGIPPR.G	925.485	0.00057	43	2.51E-06	
MKK2_MAPK20_ATG6,8142	R.NQGSGIPPR.G	925.485	-0.00021	42.46	2.84E-06	
MKK2_MAPK20_ATG6,8373	R.NQGSGIPPR.G	925.485	-0.00195	34.62	1.73E-05	
MKK2_MAPK20_ATG6,8630	R.NQGSGIPPR.G	925.485	0.00133	38.71	6.73E-06	
MKK2_MAPK20_ATG6,8893	R.NQGSGIPPR.G	925.485	0.00023	36.9	1.02E-05	
MKK2_MAPK20_ATG6,9133	R.NQGSGIPPR.G	925.485	-0.00009	33.32	2.33E-05	
MKK2_MAPK20_ATG6,9401	R.NQGSGIPPR.G	925.485	0.00101	34.41	1.81E-05	
MKK2_MAPK20_ATG6,9687	R.NQGSGIPPR.G	925.485	0.00023	39.45	5.68E-06	
MKK2_MAPK20_ATG6,9940	R.NQGSGIPPR.G	925.485	-0.00001	37.85	8.20E-06	
MKK2_MAPK20_ATG6,10202	R.NQGSGIPPR.G	925.485	0.00019	37.91	8.09E-06	
MKK2_MAPK20_ATG6,23711	R.NQGSGIPPR.G	925.485	-0.00249	38.95	6.37E-06	
MKK2_MAPK20_ATG6,8774	R.NVLSEADFLKEK.L	1392.73689	0.00314	20.81	4.15E-04	
MKK2_MAPK20_ATG6,9028	R.SGM&QASSIHGAGSAIGSTSR.M	1877.87701	0.00036	20.66	4.30E-04	15.994919 Oxidation (M)
MKK2_MAPK20_ATG6,9342	R.SGM&QASSIHGAGSAIGSTSR.M	1877.87701	-0.00003	65.86	1.30E-08	15.994919 Oxidation (M)
MKK2_MAPK20_ATG6,12286	R.SGM&QASSIHGAGSAIGSTSR.M	1877.87701	0.00135	34.19	1.91E-05	15.994919 Oxidation (M)
MKK2_MAPK20_ATG6,20447	R.SGMQASSIHGAGSAIGSTSR.M	1861.88209	-0.00036	47.06	9.84E-07	
MKK2_MAPK20_ATG6,20500	R.TLPVDPNLPR.Y	1121.63133	0.00121	32.32	2.93E-05	
MKK2_MAPK20_ATG6,20735	R.TLPVDPNLPR.Y	1121.63133	0.00293	30.4	4.56E-05	
MKK2_MAPK20_ATG6,7309	R.TLPVDPNLPR.Y	1121.63133	-0.00037	35.31	1.47E-05	
MKK2_MAPK20_ATG6,7319	R.VLSDKLDK.E	917.53021	0.00066	35.58	1.38E-05	
MKK2_MAPK20_ATG6,16607	R.VLSDKLDK.E	917.53021	-0.00073	46.45	1.13E-06	
MKK2_MAPK20_ATG6,16618	R.VLSDKLDKEVEDVNR.D	1758.9232	-0.00105	39.58	5.51E-06	
MKK2_MAPK20_ATG6,16629	R.VLSDKLDKEVEDVNR.D	1758.9232	-0.00292	24.62	1.73E-04	
MAPK20_ATG6,2241	K.ELEM&K.S	665.31743	0.00038	21.8	3.30E-04	15.994919 Oxidation (M)
MAPK20_ATG6,3803	K.EVEDVNR.D	860.41083	0.00118	41.74	3.35E-06	
MAPK20_ATG6,17534	K.FFPDSSYR.S	1018.46287	-0.00065	42.6	2.75E-06	
MAPK20_ATG6,17799	K.FFPDSSYR.S	1018.46287	-0.00043	29.81	5.22E-05	

MAPK20_ATG6,18364	K.FFPDSSYR.S	1018.46287	0.00045	20.06	4.93E-04	
MAPK20_ATG6,1640	K.GSSATPDK.G	762.36282	0.0004	82.31	2.94E-10	
MAPK20_ATG6,5	K.IENDK.V	618.30931	0.00042	20.68	4.28E-04	
MAPK20_ATG6,123	K.IENDK.V	618.30931	0.0002	28.05	7.83E-05	
MAPK20_ATG6,238	K.IENDK.V	618.30931	0.00024	27.77	8.36E-05	
MAPK20_ATG6,18951	K.IENDKVESYSITQSFNK.Q	2001.97635	-0.00058	69.64	1.09E-08	
MAPK20_ATG6,12831	K.IIPM&GSYPR.I	1049.54481	-0.00057	36.25	1.19E-05	15.994919 Oxidation (M)
MAPK20_ATG6,13087	K.IIPM&GSYPR.I	1049.54481	-0.00021	37.86	8.18E-06	15.994919 Oxidation (M)
MAPK20_ATG6,17808	K.IIPMGSYPR.I	1033.54989	-0.00007	36.04	1.24E-05	
MAPK20_ATG6,6844	K.LDKEVEDVNR.D	1216.61679	-0.00232	51.19	3.80E-07	
MAPK20_ATG6,6857	K.LDKEVEDVNR.D	1216.61679	-0.00074	49.1	6.15E-07	
MAPK20_ATG6,11467	K.LETAIEETEK.Q	1162.58374	0.00023	66.39	3.44E-08	
MAPK20_ATG6,7952	K.LKIEEEER.K	1045.55237	0.00151	38.12	7.71E-06	
MAPK20_ATG6,8040	K.LKIEEEER.K	1045.55237	0.00118	57.97	7.98E-08	
MAPK20_ATG6,14401	K.QCAVVTAELK.E	1118.58742	-0.00009	39	6.29E-06	
MAPK20_ATG6,6	K.QPTNTK.F	688.36242	0.0001	27.94	8.03E-05	
MAPK20_ATG6,18270	K.TEVSQAHLELLK.R	1367.75288	0.0006	30.2	4.77E-05	
MAPK20_ATG6,23018	R.AM&EESFVVLPPPAASVYK.C	1950.98811	-0.00157	37	9.98E-06	15.994919 Oxidation (M)
MAPK20_ATG6,23273	R.AMEESFVVLPPPAASVYK.C	1934.99319	-0.00153	39.18	6.04E-06	
MAPK20_ATG6,7453	R.DAILAK.T	630.38208	-0.00037	22.38	2.89E-04	
MAPK20_ATG6,7818	R.FKELEER.Y	950.49414	-0.00199	43.12	2.44E-06	
MAPK20_ATG6,7838	R.FKELEER.Y	950.49414	-0.00037	29.89	5.13E-05	
MAPK20_ATG6,9976	R.GSAQPDASQFGR.A	1220.56544	0.0005	54.54	5.27E-07	
MAPK20_ATG6,10439	R.KLETAIEETEK.Q	1290.67869	-0.00184	44.38	1.82E-06	
MAPK20_ATG6,16772	R.M&ENSYVM&LPK.Q	1243.5697	0.00142	56.13	1.22E-07	15.994919 Oxidation (M)
MAPK20_ATG6,17033	R.M&ENSYVM&LPK.Q	1243.5697	0.00094	54.22	1.89E-07	15.994919 Oxidation (M)
MAPK20_ATG6,19107	R.M&ENSYVMLPK.Q	1227.57478	0.00098	45.11	1.54E-06	15.994919 Oxidation (M)
MAPK20_ATG6,18824	R.MENSYVM&LPK.Q	1227.57478	0.00024	53.37	2.30E-07	15.994919 Oxidation (M)
MAPK20_ATG6,4381	R.NQGSGIPPR.G	925.485	0.00069	21.13	3.85E-04	

MAPK20_ATG6,4626	R.NQGSGIPPR.G	925.485	0.00033	42.23	2.99E-06	
MAPK20_ATG6,4862	R.NQGSGIPPR.G	925.485	-0.00019	38.89	6.46E-06	
MAPK20_ATG6,5088	R.NQGSGIPPR.G	925.485	-0.00055	32.28	2.96E-05	
MAPK20_ATG6,5297	R.NQGSGIPPR.G	925.485	-0.00045	29.04	6.24E-05	
MAPK20_ATG6,5504	R.NQGSGIPPR.G	925.485	0.00055	41.16	3.83E-06	
MAPK20_ATG6,5721	R.NQGSGIPPR.G	925.485	0.00069	33.82	2.07E-05	
MAPK20_ATG6,5971	R.NQGSGIPPR.G	925.485	0.00003	33.08	2.46E-05	
MAPK20_ATG6,6877	R.NQGSGIPPR.G	925.485	-0.00009	34.22	1.89E-05	
MAPK20_ATG6,7442	R.NQGSGIPPR.G	925.485	0.00001	22.21	3.01E-04	
MAPK20_ATG6,7679	R.NQGSGIPPR.G	925.485	-0.00027	24.99	1.58E-04	
MAPK20_ATG6,7939	R.NQGSGIPPR.G	925.485	0.00003	22.36	2.90E-04	
MAPK20_ATG6,8424	R.NQGSGIPPR.G	925.485	-0.00017	29.42	5.71E-05	
MAPK20_ATG6,8486	R.SGM&QASSIHGAGSAIGSTSR.M	1877.87701	-0.00012	78.99	6.31E-10	15.994919 Oxidation (M)
MAPK20_ATG6,11591	R.SGMQASSIHGAGSAIGSTSR.M	1861.88209	0.00045	27.98	7.96E-05	
MAPK20_ATG6,19274	R.TLPVDPNLPR.Y	1121.63133	-0.00022	31.16	3.83E-05	
MAPK20_ATG6,19473	R.TLPVDPNLPR.Y	1121.63133	0.00025	34.98	1.59E-05	
MAPK20_ATG6,7023	R.VLSDKLDK.E	917.53021	-0.00042	35.48	1.42E-05	
MAPK20_ATG6,7041	R.VLSDKLDK.E	917.53021	-0.00023	41.02	3.95E-06	
MAPK20_ATG6,15660	R.VLSDKLDKEVEDVNR.D	1758.9232	0.00105	28.12	7.71E-05	15.994919 Oxidation (M)
ATG6,2582	K.DRENNIRPDK.C	1256.63415	-0.00004	25.1	1.55E-04	
ATG6,13139	K.EFSEFANSK.D	1058.4789	-0.00111	28.06	7.82E-05	
ATG6,3668	K.EVEDVNR.D	860.41083	0.0011	38.77	6.64E-06	
ATG6,1622	K.GSSATPDK.G	762.36282	0.0006	54.64	1.72E-07	
ATG6,2750	K.IEEEER.K	804.37335	0.00019	43.45	4.52E-06	
ATG6,17898	K.IENDKVESYSITQSFNK.Q	2001.97635	0.0002	75.68	2.70E-09	
ATG6,11953	K.IIPM&GSYPR.I	1049.54481	-0.00063	33.88	2.05E-05	15.994919 Oxidation (M)
ATG6,12178	K.IIPM&GSYPR.I	1049.54481	-0.00033	41.8	3.30E-06	15.994919 Oxidation (M)
ATG6,16604	K.IIPMGSYPR.I	1033.54989	-0.00015	32.79	2.63E-05	
ATG6,6410	K.LDKEVEDVNR.D	1216.61679	0.00023	53.31	2.33E-07	

ATG6,6426	K.LDKEVEDVNR.D	1216.61679	0.00082	40.45	4.51E-06	
ATG6,7441	K.LKIEEEER.K	1045.55237	0.00064	33.97	2.00E-05	
ATG6,5716	K.QENWTK.A	805.38388	-0.00045	31.98	3.17E-05	
ATG6,17152	K.TEVSQAHLELLK.R	1367.75288	0.00028	29.49	5.62E-05	
ATG6,17999	K.VESYSITQSFNK.Q	1402.68487	0.00117	24.55	1.75E-04	
ATG6,21713	R.AM&EESFVVLPPPAASVYK.C	1950.98811	-0.00028	33.16	2.42E-05	15.994919 Oxidation (M)
ATG6,21715	R.AM&EESFVVLPPPAASVYK.C	1950.98811	-0.00159	39.36	5.79E-06	15.994919 Oxidation (M)
ATG6,7325	R.FKELEER.Y	950.49414	-0.00003	32.99	2.51E-05	
ATG6,7362	R.FKELEER.Y	950.49414	0.00002	28.41	7.21E-05	
ATG6,9239	R.GSAQPDASQFGR.A	1220.56544	0.00056	56.24	3.57E-07	
ATG6,9764	R.KLETAIEETEK.Q	1290.67869	0.00423	69.54	5.56E-09	
ATG6,9767	R.KLETAIEETEK.Q	1290.67869	-0.00135	43.57	2.20E-06	
ATG6,15687	R.M&ENSYVM&LPK.Q	1243.5697	0.0006	51.8	3.30E-07	15.994919 Oxidation (M)
ATG6,17964	R.M&ENSYVMLPK.Q	1227.57478	0.00036	35	1.58E-05	15.994919 Oxidation (M)
ATG6,17680	R.MENSYVM&LPK.Q	1227.57478	0.0008	49.59	5.50E-07	15.994919 Oxidation (M)
ATG6,4238	R.NQGSGIPPR.G	925.485	0.00031	29.36	5.79E-05	
ATG6,4434	R.NQGSGIPPR.G	925.485	0.00051	23.11	2.44E-04	
ATG6,5100	R.NQGSGIPPR.G	925.485	0.00021	24.97	1.59E-04	
ATG6,21577	R.NVLSEADFLK.E	1135.59935	0.00232	46.03	1.25E-06	
ATG6,7900	R.SGM&QASSIHGAGSAIGSTSR.M	1877.87701	-0.00027	60.49	4.47E-08	15.994919 Oxidation (M)
ATG6,18100	R.TLPVDPNLPR.Y	1121.63133	0.00098	38.31	7.38E-06	
ATG6,18285	R.TLPVDPNLPR.Y	1121.63133	0.00001	33.11	2.44E-05	
ATG6,6636	R.VLSDKLDK.E	917.53021	0.00009	26.7	1.07E-04	
ATG6,6646	R.VLSDKLDK.E	917.53021	-0.00101	42.25	2.98E-06	

Table S2. Primers used for qPCR assays.

Gene name	Accession numbers	Forward primer	Reverse primer
MAPK20	Sl07g056350	5′-CATGTGGAATGGCTGCTAAG-3′	5′-TTCGGTCATCAACACGATCT-3′
ATG1a	Sl09g011320	5′-AGTTCGGAAAGTCCCTCATC-3′	5′-ATGATAGCAGAGGCAGAACG-3′
ATG1b	Sl10g084930	5′-GGAAAGTCCCTCTTCTGCTC-3′	5′-ACTCAATTCTGGGTATGCCA-3′
ATG2	Sl01g108160	5′-GTCATTGAAGAGGCACTGCT-3′	5′-AGCTAAATCAACACGGCAAG-3′
ATG3	Sl06g034160	5′-GAGAGGAGTTGAACCCGAAG-3′	5′-CGAAGGAAGTTGACAGCAAA-3′
ATG4	Sl01g006230	5′-AATTGATCCCTCCTTGGCTA-3′	5′-GATGTGGCAGAGCTACGAGT-3′
ATG5	Sl02g036380	5′-TCAGATGGTGCTGAGATCAAG-3′	5′-ATTGTTTACCACCCATGCAA -3′
ATG6	Sl05g050390	5′-CCCATGCAGTCAAACAATTC-3′	5′-CCCTCATGCATTCAAGACAC-3′
ATG7	Sl11g068930	5′-ATTCAACGGCTAACCGTACC-3′	5′-CAAACTCAGCTTTGGCACAT-3′
ATG8a	Sl07g064680	5′-ACCGGTGATTGTTGAGAAGG-3′	5′-GCGCTGAGCTTAATCCTCTT-3′
ATG8b	Sl02g080590	5′-GGAGAGGAGGCAGTCAGAAT-3′	5′-AGTCAAATCAGCTGGGACAA-3′
ATG8c	Sl03g031650	5′-TTGGCCAATTTGTTTACGTT-3′	5′-AAAGGAATCCGTCTTCATCC-3′
ATG8d	Sl10g006270	5′-AATATCCTTCCTCCCACAGC-3′	5′-GCAGAGAGGTTTGACTGCAT-3′
ATG8e	Sl08g007400	5′-TTCGTCGACAATGTCCTACC-3′	5′-AAAGGAAACCGTCTTCATCC-3′
ATG8f	Sl08g078820	5′-GGCAATCATGTCTGCAATCT-3′	5′-TCAAAGCTACAGTTCGCTCAG-3′
ATG8h	Sl01g068060	5′-CGTGTTTGTGAATAACACCTTG-3′	5′-AGCACATGTAGAGGAACCCA-3′
ATG9	Sl04g008630	5′-ATGTGCATCCTGAAATCGAA-3′	5′-GCCTCTCGAAGAACAAGTCC-3′
ATG10	Sl09g047840	5′-GGAGAACCCTTGGCAATAGA-3′	5′-TAGTCCCACATGGATGCAAT-3′
ATG12	Sl12g049310	5′-GTGTATGTCAACAGCGCCTT-3′	5′-AAACAACCAGGAGTTCTCAGC-3′
ATG13a	Sl03g096790	5′-GATGTCGACACTTCCGATTC-3′	5′-TTGCAGATCCCTGAGAAGAG-3′
ATG13b	Sl06g072980	5′-CTGTAGGTGCCCTTGTTCAC-3′	5′-AAGCTTTGAGCTCCTCCAAT-3′
ATG18a	Sl08g006010	5′-CAGCGAGTTCACCACTATCC-3′	5′-TCCATCCAAGCCAAGAATTA-3′
ATG18b	Sl07g006120	5′-TTGAGGAGACAGCAACACCT-3′	5′-TGTTCTGATGGTTGACGTTG-3′
ATG18c	Sl01g099400	5′-GCTTTGCGCTTACAAATGAT-3′	5′-CTGCTCTATCTGCGCCTCT-3′
ATG18f	Sl12g005230	5′-TCCGAAGCAGAACTCCAAAT-3′	5′-AACCTCAGCCTCTCCACGAC-3′
ATG18g	Sl01g098430	5′-GTTCTGCTGCTGGTGAGATT-3′	5′-GAATCCTGTGGAAATGGTCA-3′
ATG18h	Sl07g064060	5′-GGCTAAATACAGTGAGCAATG-3′	5′-GTGAAAATCAGCAGCAAGAAC-3′
TDF1	Sl03g059200	5′-ATTTGCTCAGGTCTTTGCTG-3′	5′-GCAAGGGATGATGTGGAGTA-3′

MS1-like	Sl04g008420	5′-GGCTAAATCGCGATGGGTGT-3′	5′-TCAAGCAACCCTGTATCACCA-3′
MYB80	Sl10g005760	5′-GAAGATGGTTCACCATGGAA-3′	5′-TGCTGCTGCTGTTGTTGTAA-3′
DYT1	Sl02g079810	5′-TGGAGGCAATGAATGCTCTTGG-3′	5′-TGGAGGCAATGAATGCTCTTGG-3′
AMS-like	Sl08g062780	5′-TGCAGCAAATGGAGCCACAA-3′	5′-AGCCCAATGAGTTCAAAGCCT-3′
Actin	Sl03g078400	5′-TGGTCGGAATGGGACAGAAG-3′	5′-CTCAGTCAGGAGAACAGGGT-3′

Table S3. Primers used for vector construction.

Gene name	Accession numbers	Forward primer	Reverse primer	
MAPK20 (BD)	Sl07g056350	5′-CATGccatggAGATG CAGCCTGATCACCG-3′	5′-CCGgaattcCTAGTACATC CTGGACATAC-3′	Yeast two-hybrid
ATG3	Sl06g034160	5′-CGCggatccATATGGTGC TGTCACAGCTTC-3′	5′-CCGctcgagTCAGGTGCTA CTGCTACC-3′	
ATG4	Sl01g006230	5′-TCCcccgggTATGGACAA AACTGGGATGCC-3′	5′-CCGctcgagTCAAAGGAG TTGCCACTCATC-3′	
ATG5	Sl02g036380	5′-CGgaattcATGGAAAGTA AAGGAGGAGG-3′	5′-CGCggatccCTATATGGTG ATGGGTTCTTG-3′	
ATG6 (AD)	Sl05g050390	5′-CGCggatccATATGGTGA AAGGCAGCAGCGC-3′	5′-CgagctcTCAAGATTGAAA CTTGGTATTAG-3′	
ATG7	Sl11g068930	5′-CGgaattcATGGCGGATAC TGGAAAAGG-3′	5′-CCGctcgagTTATATTTCTA CACAGTCATC-3′	
ATG8a	Sl07g064680	5′-CGgaattcATGGCCAAAA GCTCCTTC-3′	5′-CCGctcgagTCAGAAGGAT CCGAAGGT-3′	
ATG12	Sl12g049310	5′-CGgaattcATGGCTGCCGA TTCTCG-3′	5′-CGCggatccTCAGCCCCA CGCCATGG-3′	

ATG13a	Sl03g096790	5′-CGgaattcATGGATTTTCA GAGTAATCC-3′	5′-CCGctcgagTCAACCCTCA CTGACTGAAC-3′	
ATG13b	Sl06g072980	5′-CGgaattcATGGATTTTCG GAGTAACTTAC-3′	5′-CGCggatccTCACGCATAG CTCCCATCCTC-3′	
MAPK20 (AD)	Sl07g056350	5′-gccatggaggccagtgaattcATGCAGC CTGATCACCGAAA-3′	5′-cagctcgagctcgatggatccCTAGTACA TCCTGGACATACCATACTGA-3′	
ATG6 (BD)	Sl05g050390	5′-atggccatggaggccgaattcATGGTGA AAGGCAGCAGCG-3′	5′-ccgctgcaggtcgacggatccTCAAGAT TGAAACTTGGTATTAG-3′	
ATG6-1	Sl05g050390	5′-gccatggaggccagtgaattcATGG TGAAAGGCAGCAGCG-3′	5′-cagctcgagctcgatggatccGGTG GAGTGAAACCCAGAATTG-3′	
ATG6-2	Sl05g050390	5′-gccatggaggccagtgaattcGAAGCA AGAAATGTTCTTAGCGAG-3′	5′-cagctcgagctcgatggatccGGTC GGAAATGCTGAGCCA-3′	
ATG6-3	Sl05g050390	5′-gccatggaggccagtgaattcGTTGGTA ACACAAATTTCCAGCC-3′	5′-cagctcgagctcgatggatccAGATTGA AACTTGGTATTAGTTGGTTG-3′	
ATG6-4	Sl05g050390	5′-gccatggaggccagtgaattcATGGTG AAAGGCAGCAGCG-3′	5′-cagctcgagctcgatggatccTTGCTTT GGCAACATAACATACG-3′	
MAPK20	Sl07g056350	5′-tacgcgtcccggggcggtaccATGCA GCCTGATCACCGAAA-3′	5′-cagtcgacgcgttgtggatccCTAGTAC ATCCTGGACATACCATACTGA-3′	Split-luciferase assay
ATG6	Sl05g050390	5′-acgggggacgagctcggtaccATGGTG AAAGGCAGCAGCG-3′	5′-cagtcgacgcgttgtggatccAGATTGAA ACTTGGTATTAGTTGGTTG-3′	
MAPK20	Sl07g056350	5′-GGCCatttaaatATGCAGCCTGAT CACCGAAAG-3′	5′-GCtctagaGTACATCCTGGACA TACC-3′	BiFC
ATG6	Sl05g050390	5′-GCtctagaATGGTGAAAGGCA GCAGCGC-3′	5′-CGCggatccAGATTGAAACTTG GTATTAG-3′	
mCherry		5′-ttacaattaccatggggcgcgccATGGTG AGCAAGGGCGAGG-3′	5′-tcttagccatTCGCGACTTGTACA GCTCGTC-3′	
ATG8f	Sl08g078820	5′-caagtcgcgaATGGCTAAGAGCT CATTCAAGCA-3′	5′-gactctagggactagttaattaaCTACAG TTCGCTCAGGACCCC-3′	
MAPK20	Sl07g056350	5′-TTggcgcgccATGCAGCCTGAT	5′-GGggtaccGTACATCCTGGACA	Overexpre

		CACCGAAA-3′	TACCATACTGA-3′	ssing
MKK2^{DD} (HIS)	Sl03g123800	5′-gccatggctgatatcggatccATGCGACCAGCCGCCAAC-3′	5′-gtggtggtggtggtgctcgagTAGAAGAGGAGGAAAAATGAGGAGG-3′	Pull-down and *vitro* phosphorylation
		5′-gtcattgcaaggatccatatcTTGTGCCAGAACTCTTGAGACCC-3′	5′-tggatccttgcaatgacTCAGTGGGTACCATCGCTTACA-3′	
ATG6 (GST)	Sl05g050390	5′-ccgcgtggatccccggaattcATGGTGAAAGGCAGCAGCG-3′	5′-gtcacgatgcggccgctcgagTCAAGATTGAAACTTGGTATTAGTTGG-3′	
MAPK20 (HIS)	Sl07g056350	5′-gctgatatcggatccgaattcATGCAGCCTGATCACCGAAA-3′	5′-ctcgagtgcggccgcaagcttCTAGTACATCCTGGACATACCATACTGA-3′	
MAPK20 (MBP)	Sl07g056350	5′-gagggaaggatttcagaattcATGCAGCCTGATCACCGAAA-3′	5′-caggtcgactctagaggatccGTACATCCTGGACATACCATACTGAAC-3′	
MKK2^{DD} (Flag)	Sl03g123800	5′-ttacaattaccatggggcgcgccATGCGACCAGCCGCCAAC-3′	5′-gtccttgtagtcagaggtaccAGAAGAGGAGGAAAAATGAG-3′	Co-IP and IP-MS
		5′-gtcattgcaaggatccatatcTTGTGCCAGAACTCTTGAGACCC-3′	5′-tggatccttgcaatgacTCAGTGGGTACCATCGCTTACA-3′	
MAPK20 *(MYC)*	Sl07g056350	5′-catatggggctgcaggaattcATGCAGCCTGATCACCGAAA-3′	5′-gggactagaactagtggatccCTAGTACATCCTGGACATACCATAC-3′	
ATG6 (HA)	Sl05g050390	5′-ttacaattaccatggggcgcgccATGGTGAAAGGCAGCAGCG-3′	5′-aacatcgtatgggtaggtaccAGATTGAAACTTGGTATTAGTTGGTTG-3′	
ATG6 (GFP)	Sl05g050390	5′-ctctcgagctttcgcgagctcATGGTGAAAGGCAGCAGCG-3′	5′-gcccttgctcaccatggatccAGATTGAAACTTGGTATTAGTTGGTTG-3′	
